# Habitat filtering more than microbiota origin controls microbiome transplant outcomes in soil

**DOI:** 10.1093/ismejo/wraf162

**Published:** 2025-08-01

**Authors:** Senka Causevic, Janko Tackmann, Vladimir Sentchilo, Lukas Malfertheiner, Christian von Mering, Jan Roelof van der Meer

**Affiliations:** Department of Fundamental Microbiology, University of Lausanne, 1015 Lausanne, Switzerland; Department of Molecular Life Sciences and Swiss Institute of Bioinformatics, University of Zürich, 8006 Zürich, Switzerland; Department of Fundamental Microbiology, University of Lausanne, 1015 Lausanne, Switzerland; Department of Molecular Life Sciences and Swiss Institute of Bioinformatics, University of Zürich, 8006 Zürich, Switzerland; Department of Molecular Life Sciences and Swiss Institute of Bioinformatics, University of Zürich, 8006 Zürich, Switzerland; Department of Fundamental Microbiology, University of Lausanne, 1015 Lausanne, Switzerland

**Keywords:** microbiome, coalescence, transplants, habitat filtering, microbiota origin, soil, lake

## Abstract

Human activities cause a global loss of soil microbiome diversity and functionality. One way to reverse this trend is through microbiota transplants, but the processes determining merger outcomes are not well understood. Here, we investigated the roles of habitat filtering and microbiota origin on microbiome development upon mergers, with the hypothesis that native strains are better adapted to their own habitat and will outcompete non-native ones in niche colonization. To test this, we contrasted community development in soil microcosms between two taxa-diverse microbiota originating from either topsoil [SoilCom (SC)] or freshwater lake [LakeCom (LC)], and a defined mixture of 21 soil bacteria (SynCom). When inoculated separately, SC and LC showed similar taxa and colonization patterns contributing to community growth and decline within the soil microcosms. SynCom transplants to either SC or LC under renewed growth conditions permanently altered their community trajectories, and slightly further converged their taxa compositions. Levels of SynCom members in both resident backgrounds decreased from initial 50–80% to below 1% within 2 months. Merged as well as non-merged communities resembled natural soils in comparison to over 81 000 publicly available soil, sediment, and lake microbiomes. Our results show that habitat filtering is dominant over microbiota taxa origin in determining transplant outcomes. Even though the proliferation of SynCom transplants remained limited, their capacity to influence community merger trajectories long term opens new paths for soil microbiome engineering.

## Introduction

Soils encompass staggering microbial diversity and biomass, which sustain diverse plant and animal life [1, 2]. Microbiomes largely contribute to soil fertility by transforming and building organic matter, establishing aggregate formation, and facilitating nutrient provision to plants [3, 4]. Collectively, soil microbiomes carry out crucial roles in the major planetary biogeochemical cycles [5, 6]. Soil microbiome diversity, organic matter, and structure are threatened world-wide as a result of agricultural practices, pollution, poor land management, erosion, and urbanization [7, 8]. Degraded soil structure and microbiome diversity loss affect food production [8], ecosystem stability, and climate [9]. There is thus an urgent need to restore degraded soil, but this can only be achieved with detailed understanding of the processes underlying assembly of taxa-complex soil microbiomes, and the causes leading to dysfunctional system states.

One restoration approach involves regeneration of the soil microbiome [10–12]. Single inoculants with beneficial character in combination with prebiotic substrates can be used for this [13, 14], but these are unlikely to overcome the multiple factors, regional properties, and scales of soil loss (globally one third of soils are considered degraded) [15, 16]. Alternatively, strain consortia with defined functional guilds could be applied, but there is insufficient knowledge to ensure their efficacy. Similar to clinical gut microbiome interventions [17, 18], one could also imagine a reset of the impacted soil microbiome followed by habitat recolonization from a community transplant of a healthy site. In contrast to the gut, where antibiotics can be applied to diminish the abundance of resident bacteria before adding the transplant, resetting a soil microbiome is not achievable at scale. Hence, any soil transplant would have to compete with the resident microbiota of the impacted soil, leading to microbiota coalescence [19].

In the context of soil, several studies have shown positive effects of transplants on plant disease suppression, drought tolerance, or climate tolerance of trees [20–22]. The processes underlying transplant mergers with resident microbiota are unknown, and there is little theory to predict coalescence outcomes and resulting taxa changes [23]. To build testable hypotheses, it is crucial to use controlled systems with reproducible communities, which limit the confounding effects of biotic and abiotic variables present in natural soils. We recently showed how defined (from cultured isolates) and taxa-diverse undefined soil microbiota (from washed microbial cell fraction of native soil) can be grown in microbe-free soil [24]. Both inocula mature reproducibly in weeks to months into compositions with clear soil community signatures [24], making them ideal resources to systematically investigate coalescence processes. The main goal of our work was to study the effects of two processes that we hypothesized could be underlying community mergers: (i) habitat filtering (i.e. nutrients and spatial niches selecting taxa succession based on their functional traits) and (ii) microbiota origin (i.e. the starting taxa composition, its fundamental metabolic capacities [25], and its potential for forming interspecific interactions).

**Figure 1:**
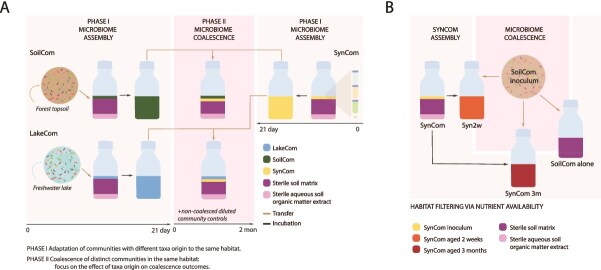
Experimental designs for community development and coalescence in defined pristine soil microcosms. (A) Phase I: testing the effect of habitat on the formation of communities starting from soil-washed inoculum (SC), filtered freshwater lake inoculum (LC), or a 21-membered culturable synthetic soil strain mixture (SynCom, all seeded at ca. 10^6^ cells per gram) in soil microcosms (4 biological replicates per condition). Phase II: set-up with soil habitat-adapted communities to test taxa origin-effects through coalescence. SC or LC are mixed with SynCom in a 1:1 soil-weight ratio and supplemented with 8-fold sterile soil with fresh supply of soil organic extract in new microcosms. In addition, as controls, each community is diluted non-coalesced in new microcosms as well (as before, four biological replicates per condition). (B) Testing coalescence as a function of habitat nutrient availability. Two weeks (Syn2w) and 3 months (Syn3m)-aged SynComs in soil microcosms are inoculated or not with fresh SC inoculum (washed cell suspension only) to a density of 10^5^ cells per g and incubated for 1 month. All steps were conducted with *n* = 4 replicate microcosms.

To disentangle the effects of habitat and microbiota origin, we designed a two-phase experiment (Fig. 1A). First, we cultured taxa-diverse microbiomes from different origins (freshwater lake and forest top soil) in sterile soil microcosm habitats for 21 days following previous methods [24]. As freshwater lake receives soil run-off, its microbiota carries taxa similar to soil that could develop into a soil microbiome under habitat selection, but still maintains distinct taxa memberships and niche preferences [26, 27]. Considering SC and LC origins were non-native for their new (soil microcosm) habitat, we expected that if habitat filtering was driving community development, we would see similar taxa demography and converging compositions over time.

In the second phase (Fig. 1A), we aimed to detect more precisely the influence of microbiota origin in coalescence experiments, while maintaining the same soil microcosm habitat. For this, we cultured a defined community (SynCom) consisting of 21 isolates covering four major bacterial phyla originating from the same site as the SC inoculum, that had previously been shown to reproducibly colonize fresh soil to high density [24]. Coalescence was induced by mixing the same weight of the three composed soil-habitat adapted communities (from Phase I) into fresh sterile soil microcosms to restart community growth (SC or LC soil portion mixed with that of SynCom; monodiluted SC, LC, or SynCom as controls; Fig. 1A). Our assumption was that if microbiota origin would dominate, we would find deviating trajectories for the coalesced communities compared to their non-merged controls, particularly for the merger of LC and SynCom, because SynCom taxa are foreign to LC. This would give the starting community a different set of inherent metabolic capacities that could potentially lead to other emerging interspecific interactions and changing developmental trajectories. If habitat filtering would be dominant, we would find convergence of coalesced and control non-merged communities, because despite having different functional potential, the habitat would select for similar functional traits. Finally, we tested the invasion of SC inoculum into soil-grown SynComs at different stages of maturation (2 weeks and 3 months, Fig. 1B) to understand whether nutrient niche availability is important for merger outcomes.

## Materials and methods

### Preparation of resident and transplant communities

Three communities in this study were SC, LC, and SynCom. SC was started from inoculum recovered by washing cells of forest topsoil (−5 to −15 cm; location: GPS 46.52126, 6.57864) as described previously [24] (Supplementary methods). The cell density of the suspension was measured with flow cytometry, adjusted to 10^7^ cells ml^−1^ using soil extract (SE; Supplementary methods), after which 10 ml was used to inoculate soil microcosms.

LC originated from the freshwater Lake Geneva sampled at Port de Pierrettes (GPS 46.516937, 6.579464; February 2023), which was filtered and resuspended in filter-sterilized and autoclaved lake water (Supplementary methods). The resulting cell suspension was centrifuged for 10 min at 3220 × g (Eppendorf A-4-62 Swing Bucket Rotor), the cell pellet was resuspended in SE, and quantified with flow cytometry to obtain a density of 10^7^ cells ml^−1^ for inoculation of the soil microcosms.

SynCom was assembled from 21 cultured soil isolates (Table S1, Supplementary data) obtained from the same location and soil type as SC [24]. Strains were recovered from −80°C stocks by plating on R2A (DSMZ GmbH) and incubated for 1 week at 23°C. Grown colonies were washed from the plates, the turbidity of cell suspensions was measured using an Ultrospec 500pro spectrophotometer (Amersham Biosciences), and diluted to an OD_600_ = 1. Equal volumes of individual SynCom member suspensions were mixed together and then centrifuged at 3220 × g (Eppendorf A-4-62 Swing Bucket Rotor) for 10 min. The supernatant was discarded, and the cell pellet was resuspended in SE to reach OD_600_ = 0.01 (equivalent to 10^7^ cells ml^−1^).

### Soil microcosm preparation

Soil microcosms consisted of 90 g sterilized riverbank material in 500-ml capped Schott glass bottles. Briefly, 10 kg material was sampled in the 0–10 cm horizon, dried for 2 weeks at ambient air, and then twice autoclaved. Upon inoculation, soil microcosms received 10 ml SE, providing thus ca. 10% gravimetric water content. For soil-to-soil microcosm transfers, the proportions of soil matrix, SE, and the inocula were adjusted accordingly to maintain constant water content. Each condition was carried out in four replicates.

### Microcosm inoculation and coalescence

Sterile soil microcosms received 10 ml of SC, LC, or SynCom suspensions in SE to achieve starting densities of ca. 10^6^ cells g^−1^. Inoculated microcosms were mixed on a roller-board and incubated upright in the dark at 23°C. Mixing was repeated before sampling at the start and 3, 7, 14, and 21 days post-inoculation.

Coalescence was initiated by combining individual communities (SC, LC, or SynCom) grown separately for 1 month in soil microcosms (Fig. 1A). In this case, glass bottles were filled with 80 g of sterile soil matrix and 8 ml of sterile SE, to which either 10 g of SC and 10 g of SynCom, or 10 g of LC and 10 g of SynCom were mixed. As controls, 10 g of each individual community was inoculated into 80 g of sterile soil matrix with 8 ml of SE. The resulting coalescence microcosms were again mixed, incubated as before, and sampled at start, and after 3, 7, 21, and 60 days. The aging effect on coalescence (Fig. 1B) is described in Supplementary methods.

### Microcosm sampling

Sampled aseptically (Sterileware spatulas, SP Bel-Art), 10 g of microcosm material was transferred into a 50-ml sterile polypropylene tube. Cells were extracted from soil by mixing with 10 ml of pyrophosphate solution (Supplementary methods) and vortexing for 2 min at maximum speed on the Vortex-Genie 2 (Scientific Industries, Inc.) with vertical adaptor (SI-V525). After 2 min of sedimentation, the supernatant was transferred to a 15 ml tube, from which two aliquots of 250 μl were removed for community size enumeration. The remainder was centrifuged at 3220 × g for 7 min in an Eppendorf A-4-62 swing bucket rotor, and the resulting cell pellet was resuspended in 2 ml soil buffer, transferred to 2-ml tubes (Eppendorf), harvested by centrifugation at 7000 × g for 7 min, and stored at −80°C until deoxyribonucleic acid (DNA) isolation.

### Community size determinations

Community sizes were measured in the obtained cell suspensions by flow cytometry, colony formation (CFU counting), or by quantified DNA concentration.

For flow cytometry counting, an aliquot of cell suspension from each sample was filtered using a 40–μm strainer (Falcon) and fixed with an equal volume of 4 M sodium-azide (Sigma-Aldrich). Two technical replicates per sample were stored at 4°C. The following day, one technical replicate was stained with SYBR Green I according to the Thermo Fisher protocol; the other was unstained. Events in 10 μl sample were counted with a CytoFLEX Flow Cytometer (Beckman Coulter, 10 μl min^−1^ flow rate). The difference in the FITC-H channel signal between stained and unstained, and the comparison to a stained sample from a non-inoculated microcosm was used to define the cell gate (Fig. S1).

For CFU counting, the cell suspensions from each sample were serially diluted using soil buffer, and aliquots of the dilutions (10 μl) were dropped on R2A plates in four technical replicates. After drying, plates were incubated in the dark at room temperature. Colonies per droplet were counted using a 10× magnifying binocular two days post-plating. Samples from non-inoculated microcosms, soil buffer (used for sample dilution), and pyrophosphate solution used for cell extraction were controlled for sterility.

### Deoxyribonucleic acid isolation and amplicon sequencing

DNA was extracted from −80°C-stored cell pellets using the DNeasy PowerSoil Pro kit (Qiagen) according to the manufacturer’s protocol. DNA concentrations were measured using Qubit dsDNA BR kit (Invitrogen), and purified DNA was stored at −20°C.

Libraries for the 16S ribosomal ribonucleic acid (rRNA) V3–V4 gene amplicon sequencing were prepared according to the Illumina protocol (https://support.illumina.com/content/dam/illumina-support/documents/documentation/chemistry_documentation/16s/16s-metagenomic-library-prep-guide-15044223-b.pdf), from 10 ng purified DNA per sample. Libraries were indexed with Nextera XT Index Kit v2 set A and B (Illumina). PCR products were purified using 0.85 v CleanNGS beads (CleanNA) and quantified as above. Following equimolar pooling and final bead purification, the multiplexed libraries were paired-end sequenced on a MiSeq System (Illumina) using the 300-cycle MiSeq reagent kit v3 and 30% PhiX-DNA spike-in control at the University of Lausanne Genomics Technology Facility.

Sequencing data were cleaned and analysed with QIIME2 (version: 2021.8) within a Singularity container (version 3.8.5) on UNIX. Unique amplified sequence variants (ASV) were taxonomically referenced to SILVA (version 132). Feature and taxonomy tables, reference sequences, and phylogenetic tree were joined in a *phyloseq* object (R package, version: 1.40.0) for diversity analysis. SynCom ASVs within coalesced samples were identified based on ASVs detected in the SynCom alone controls.

To estimate the number of genome equivalents from the quantified DNA concentration (as indirect comparison to the flow cytometry cell counting), we assumed a mean genome weight per cell of 0.00292 pg for LC and 0.00511 pg for SC, calculated from estimated average genome sizes for lake and soil microbiomes [28, 29].

### MicrobeAtlas comparison

All sample sequences were compared with a global background of soil, sediment, and lake water communities from the MicrobeAtlas database (https://microbeatlas.org). Raw 16S rRNA gene amplicon reads from all samples were standardized and quality-filtered using a custom C++ program internal to the MicrobeAtlas pipeline, and mapped using MAPseq 1.2.6 [30](16S rRNA gene reference database: MAPref v2.2; all other parameters kept at default) to obtain 97%-level OTU count tables compatible with MicrobeAtlas (Supplementary methods).

### Data analysis

Data were processed and statistically analysed using MATLAB (v.2021b) and R 4.0 (R Core Team, 2019) on RStudio (version 2022.2.3.492) (Supplementary methods).

Absolute taxa abundances were calculated by multiplying the total community size (measured with flow cytometry) with the relative abundance of that taxon obtained with amplicon sequencing in the same sample. Community demography in SC and LC in Phase I and Phase II was estimated from the per-genus individual absolute abundance differences between day 1–3, day 3–7, and day 7–21. Taxa were grouped by being common to both community types or exclusive to either, and further by increasing or decreasing in both, or showing opposite behavior. The summed contribution of the taxa groups to overall community growth or decline was then quantified from the total taxa abundances at each time point.

ASV-level shifts from mergers with SynCom were evaluated by pairing abundances of common ASVs found in the following comparisons: LC+SynCom vs. LC, SC+SynCom vs. SC, and LC or SC replicates by themselves in all possible paired combinations (Supplementary methods).

## Results

### Soil and lake water inocula develop into distinct soil-like communities in the same pristine soil habitats

We cultured two taxa-diverse communities in sterile soil microcosms comprised of non-native material for both inocula origins (Fig. 1A, Phase I). The first type was inoculated with the pool of microbial cells washed from forest topsoil (−5 to −15 cm, named SC), whereas the second contained cells filtered from a freshwater lake (0 to −1 m, LC). A total of 27.7% of genera in the freshwater lake inoculum overlapped with those in the soil inoculum (singletons and non-assigned taxa removed), whereas 1% of the detected ASVs from amplified V3–V4 regions of the 16S rRNA genes were shared.

**Figure 2:**
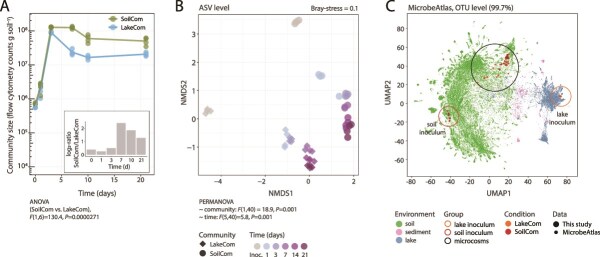
Soil habitat colonization by soil or freshwater lake inoculum and community convergence. (A) Community development over time in the soil microcosms of SC (in green) and LC (in blue), estimated total community counts by flow cytometry. Lines connect the means of the four biological replicates per condition (presented as dots). *P*-values relate to the effect of community type on attained population sizes, as obtained with two-way repeated measures ANOVA on ranked values (Supplementary data). (B) ASV-level community compositional changes over time and per biological replicate (relative abundances from sequence data), compared using Bray–Curtis distances presented with NMDS ordination. Time progression depicted with color transition from gray to dark purple. (C) Uniform manifold approximation and projection (UMAP) for dimension reduction of SC and LC inocula (red and orange circle, respectively), and soil microcosm samples (black circle) on all publicly available soil (green dots), sediment (pink dots), and lake (blue dots) microbiome compositions from the MicrobeAtlas database. UMAP comparison in MicrobeAtlas is carried out within its own consistent compositional assignment method (mapseq), and we use here a 97% similarity cutoff, which is equivalent to genus OTU level.

Community sizes measured in soil-washed cell suspensions either from culturable (colony forming units, CFU), total (flow cytometry) cell counts, or from DNA concentrations, increased rapidly during the first three days to more than 100-fold their inoculated densities, indicating active growth of both inocula (Figs 2A and S2A and B). The three methods for community size determination gave comparable counts, except for lower CFU counts in case of LC (Figs 2A and S2A and B), likely due to limited culturability on the R2A medium. Both communities reached maximum densities between day 3 and 7, after which their sizes declined and stabilized until day 21. LC sizes at day 7 and 21 remained 2–4-fold lower than those of the SC (Figs 2A and S2A and B insets).

Despite showing lower inoculum diversity, LC microbiota in soil microcosms developed higher detectable taxa diversity than SC after 21 days of incubation (Fig. S2C, two-way repeated measures ANOVA on ranked values for Shannon Index community and time effect, *F*(5,25) = 22.9, *P*-value = 1.31 × 10^−8^, post hoc *t*-test at day 21, *P-*value = .0002, Supplementary data). The compositions of LC and SC converged during habitat colonization (Fig. 2B, family level in Fig. S2D), and the proportion of shared ASVs increased to 12% after 21 days. Still, community compositions remained overall significantly different [Fig. 2B; permutational multivariate analysis of variance (PERMANOVA), *P-*value = .001 for community type and time effect].

Both types of soil-grown microbiota showed distinct soil, rather than lake compositional signatures in a comparison to a global background of 81 627 soil, lake, and sediment communities from MicrobeAtlas [31] (Figs 2C and S2E, >97% OTU similarity level). SC and LC compositions clustered separately from their inocula, each of which resembled the microbiota compositions typical of their origins. These results indicated that both inocula, regardless of origin develop into soil-like communities when grown in the same (non-native) pristine soil habitats. This demonstrated the strong role of habitat filtering in community development, selecting for growth of taxa with similar functional traits.

### Common freshwater lake and soil genera drive community growth and decline within the soil habitat

To understand which taxa and developmental stages were responsible for the observed growth trajectories of SC and LC, we analysed absolute taxa fluctuations over time. These were inferred by multiplying taxa relative abundance (sequencing data) with the quantified total community size at each time point (total flow cytometry counts, as these are independent of culturing bias, Fig. S3A). This calculation is justified as both measures are derived from the same cell suspensions washed from the soil. We acknowledge that some bias is inherent to these abundances because of differences in DNA isolation and PCR amplification efficiency of 16S rRNA gene regions.

**Figure 3:**
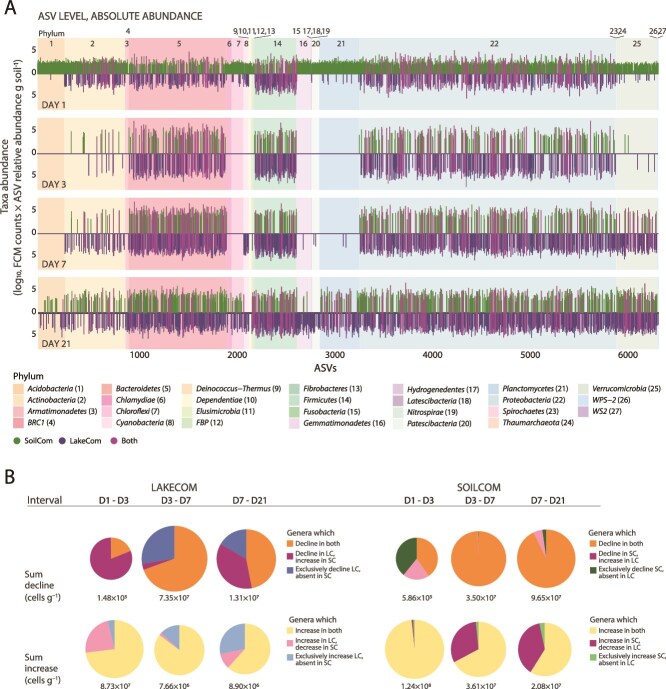
Compositional differences during development of SC and LC, and their contributions to net growth and decline. (A) Absolute ASV-levels in SC and LC at each sampled time (bars are the means of 4 biological replicates per community, log_10_-transformed values). Green bars, ASVs from SC; blue bars, LC; magenta bars, overlapping ASVs. Shaded colored background, phyla attribution of the various ASVs according to the color legend. (B) Contribution of taxa groups defined in Fig. S3B to the community increase or decline in each time interval indicated. Circle size is proportional to the absolute increase or decline of the community in the time interval (as cells g soil^−1^). Pie slices colored by taxa group behavior according to color legend below. Remark, for example, the high contributions of common genera in LC and SC to the community increase (yellow, lower panel) and decline (orange, top panel)

The absolute ASV-abundances over time in both communities showed successions of faster (e.g. in the *Proteobacteria*, *Bacteroidetes*, and *Firmicutes* phyla) and slower growing taxa (e.g. *Actinobacteria* and *Verrucomicrobia*, Fig. 3A). LC showed growth of taxa after 21 days in phyla that were underrepresented in its inoculum, but which are typical for soils (e.g. *Acidobacteria*, *Gemmatimonadetes*, and *Planctomycetes*). Only LC developed detectable numbers of *Cyanobacteria* and *Patescibacteria* members after 21 days, despite these being present in the SC inoculum as well (Fig. 3A).

We summed ASV abundances per genus and compared the summed abundance differences across three time intervals (day 1–3, 3–7, and 7–21) for both shared and exclusive genera in LC and SC (Fig. S3B). The net contribution per time interval of the genera in each comparison group to the community size increase or decline was then calculated (Fig. 3B). The early time interval (day 1–3) is characterized by the highest net growth, but this involves only 30 genera in common to LC and SC (Fig. 3B), whereas a multitude of genera decline (Figs 3B and S3C). The next interval (day 3–7) shows a drastic decline in both communities, which is less compensated by growth of genera in LC than in SC. This decline is largely due to only a dozen common genera belonging to *Gammaproteobacteria* (34% decrease in SC and 81% in LC size; mostly comprising *Acinetobacter* and *Pseudomonas*) and *Bacteroidetes* (e.g. *Flavobacterium* and *Pedobacter*, Figs 3B and S3C). In the last interval (day 7–21) *Gammaproteobacteria* further decreased (43% in SC and 35% LC). This decline in SC consisted entirely of common genera with LC. LC showed an additional decline of 30 to 55% in size attributable to a variety of other, exclusively LC genera, or genera differentially reacting in LC than in SC (Fig. 3B). The *Gammaproteobacteria* loss was accompanied by an increase of *Alphaproteobacteria* genera that contributed 26% of the summed community growth between day 3 and 7, and 15% (SC) to 26% (LakeCom) between day 7 and 21 (Fig. S3C). Growth between day 3 to 7 and 7 to 21 was largely caused by common genera, and there was no evidence for genera exclusive for the SC contributing more than 5% to community growth. However, around one-third of growing fraction in SC consisted of genera that showed a decline in LC (Fig. 3B). LC showed higher variation in declining and increasing genera than SC, which may be a sign of the larger shift associated with habitat adaptation. This analysis confirmed the dominant filtering role of the soil habitat in favoring growth and decline of common taxa in both communities, which, despite their different origins have similar functional traits. However, the stronger observed variation of LC taxa must originate from compositional differences in lake compared to soil inoculum (i.e. microbiota origin effect).

### Transient survival and proliferation of the synthetic soil community upon coalescence

Following the first phase of habitat colonization, we obtained compositionally distinct microbiomes (LC and SC) with soil community signatures, now within the same habitat (the microcosms). This enabled us to more clearly delineate the effect of microbiota origin in community development upon merging. To initiate coalescence, we diluted the mature LC and SC in sterile soil microcosms at 1:10 w:w ratio, mixing them or not with the same soil weight of the grown synthetic community of 21 culturable members (SynCom, Fig. 1A, Phase II; SynCom assembly shown in Fig. S4).

**Figure 4:**
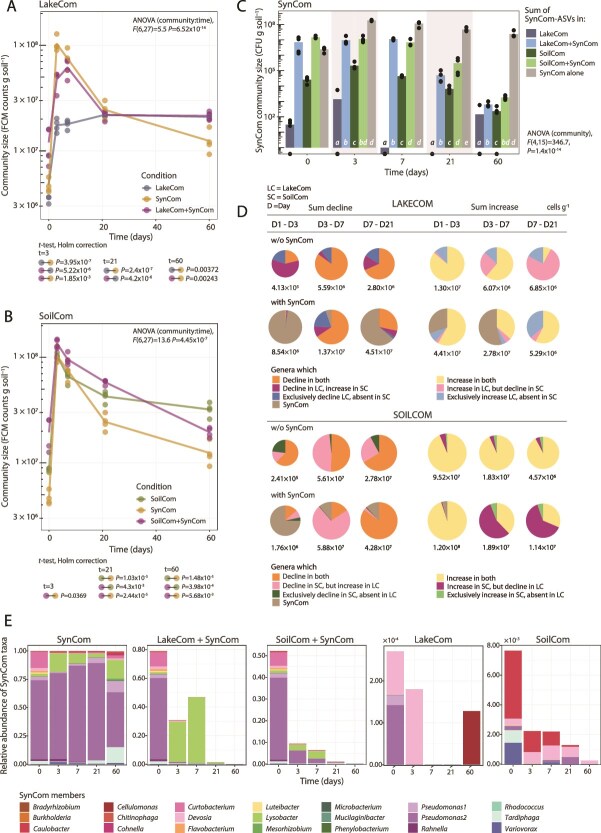
Transient SynCom survival upon coalescence with LC and SC affecting overall community growth. Flow cytometry counts of merged and individual community sizes, for LC (A) and SC mergers (B). Individual biological replicate values appear as circles, with lines connecting the means. Blue color, LC alone, green color, SC alone; magenta, merger with SynCom; yellow, SynCom alone. Two-way repeated measures ANOVA on ranked values tests for community and time effects. Post hoc *t*-tests show paired comparisons at specific time points (Holm’s correction, *P*-values only shown if <0.05). Connected circles and colors below panels of the plot indicate the comparison pair. (C) SynCom summed community sizes across all conditions. Community size represented as total viable counts per gram, multiplied, and summed from the relative SynCom ASV abundances and the total community CFU counts in each sample and condition. Black circles show biological replicate values, and bars show their means (*n* = 4 biological replicates). Bar colors represent community type as per the panel legend. Letters show significance groups, for each time point shown separately (except *t* = 0), obtained with pairwise *t*-tests (Holm’s correction) following a significant *P*-value of repeated measures ANOVA on ranked values. (D) ASV-grouped genera contributions to overall community growth and decline in three time intervals as in Fig. 3B. (E) Mean relative abundances of the SynCom members (within the summed fraction displayed in c) as proportion of the total community composition. Colors on the stacked bar plot indicate SynCom taxonomic membership as per the legend shown below the panel. The proportional advantage given to SynCom at day 0 in LC +SynCom merger (ca. 80%) is caused by their Phase I distinct population sizes, and which occurred by mixing equal soil weights of both communities as inoculum for Phase II condition (Fig. 1A).

As in the incubation phase, growth was observed for all communities during the first three days after mixing into the fresh soil microcosms (Fig. 4A and B). The total community sizes of the SynCom+ LC surpassed that of the non-merged LC at days 3 and 7, but this effect leveled off after day 20 (flow cytometry, Fig. 4A). SynCom mergers with SC also resulted in higher community sizes than SC by itself, but the effect was less prominent than for LC mergers (Fig. 4B). Eventually, the sizes of SC+SynCom and SynCom by itself decreased to below those of SC alone (Fig. 4B).

Although the sizes of SynCom-merged communities were higher than of their non-merged controls at day 3 and 7 (Fig. 4A and B), SynCom within those communities showed no net growth, whereas the SynCom on its own did (Fig. 4C). The absolute increase and decline of common and exclusive genera within three time intervals (i.e. day 1–3, 3–7, and 7–21) showed notable differences among the merged and non-merged, and between LC and SC communities (Fig. 4D). Although SynCom members showed no net growth within SC mergers, some of their populations did increase within LC up to day 7 (Fig. 4D), almost entirely due to temporal blooming of the SynCom-member *Lysobacter* (Fig. 4E). Measurements at day 60 showed further SynCom decrease to levels only slightly higher than their natural abundance in the soil (Fig. 4C, post hoc *t*-test after significant two-way repeated measures ANOVA on ranked values; Supplementary data), but with different compositions among the systems (Fig. 4E, non-merged communities can have the same ASVs as the SynCom).

The contributions of common and exclusive genera (excluding SynCom) in SC and LC mergers to overall growth and decline were different than in non-merged communities (Fig. 4D). Fast expectation–maximization microbial source tracking (FEAST), that quantifies the presence of the source communities at each time point, suggested that both SynCom and either LC, or SC can be detected in the mergers up to day 7, after which the fractions of SynCom in the mergers decline to below detection (Fig. S5A and B). An “Unknown” fraction, unattributable to either source appeared over time, reflecting the compositional shift in the mergers following the transplant (Fig. S5A and B). These results showed that SynCom only transiently proliferates within the resident soil communities upon merging, which is still sufficient to alter the coalescence trajectories compared to non-merged controls.

### Coalescence is characterized by distinct and permanent taxa changes, but resulting communities remain soil-like

**Figure 5:**
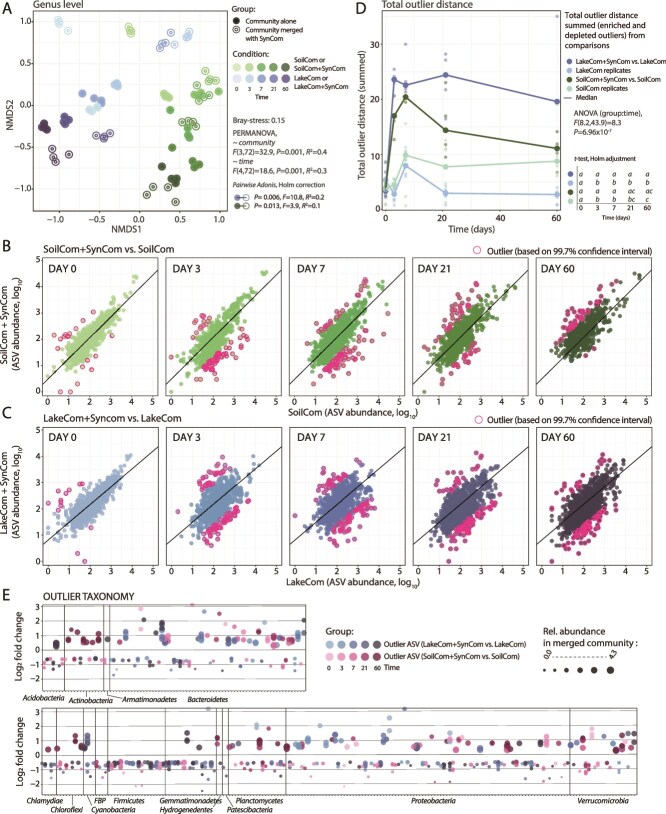
Effects of SynCom merger on LC and SC compositional trajectories. (A) NMDS ordination of Bray–Curtis distances from pairwise grouped ASV-to genus level sample comparisons among merged and non-merged communities. Circles indicate individual replicate communities, with color darkness representing time progression (color legend on the right of the panel). Filled circles, non-merged SC (green) or LC (blue), non-filled circles for merged communities. PERMANOVA test for effects of community type and incubation time. Connected circles on the right side of the plot show pairwise post hoc comparison between both merged communities (non-filled circle), and their non-merged controls (filled circle; blue, LC; green, SC; Supplementary data). Taxa displacement analysis for SynCom merger with SC (B) and LC (C). Circles show paired log_10_-transformed ASV abundances in SynCom-merged (but SynCom reads removed) vs. non-merged controls (*n* = 4 biological replicates, paired randomly). ASV abundances paired from equalized datasets, all randomly subsampled to 100 000 reads. Individual plots show outlier ASVs (magenta circles) at each time point, defined as having a residual distance to the *t* = 0 regression line (black line in plots) above the 99.7% confidence interval (derived from the residual dispersion of the t = 0 paired data). (D) Distance of outliers (identified in between-replicate, non-merged community, and merged vs. non-merged comparisons) to expected values is summed across time, per replicate (individual dots), and comparison group. Median total distance across time is shown using lines (colors indicated on the figure). Comparison within replicates of non-merged communities contained all possible replicate combinations (in total 6), whereas the merged vs. non-merged comparison had 4 replicate combinations. The effect of time and comparison group was tested using two-way repeated measures ANOVA on ranked values. Letters on the right side of the figure show per-comparison similarities based on pairwise post hoc *t-*tests (full report in supplementary data) (E) phyla attribution of ASV outliers detected in SynCom-merged LC (hues of blue, darker are later time points) or SC (hues of magenta). Circles represent the mean log_2_-fold change of the respective outlier ASV abundance in the merged vs. its non-merged community control, with circle size corresponding to its relative abundance in the merged community background (as per legend on the panel top).

To analyse community compositional differences upon coalescence, we compared taxa abundances in merged and non-merged communities. Given the low ASV-overlap of SC and LC, we focused on genus level for non-metric multidimensional scaling ordination of merged and unmerged LCs and SCs (Fig. 5A). Communities followed the same trajectory directions in NMDS, but with statistically significant separation of LCs and SCs, and by time (Fig. 5A, PERMANOVA *P-*value *=* .001). The effects of coalescence on compositional variation for both LC and SC were clearest from day 0 to 7 (when the SynCom proportion is still relatively high, Fig. 5A), but signatures for each of the merged communities vs. their non-merged control were significantly distinct across time (Fig. 5A, pairwise *adonis*, *P-*value < .05; Supplementary data). Later timepoints of LC +SynCom located closer to SC+SynCom, than the same timepoints of LC to SC (Fig. 5A). These results indicated that, when adapted and propagated in the same habitat, the development trajectory keeps a strong signature of microbiota origin. Coalescence with SynCom leads to a minor convergence of both starting communities.

To describe the level of convergence caused by SynCom coalescence, we paired common ASV abundances per time point between merged and non-merged communities, and quantified the residual variation (SynCom ASVs excluded, Supplementary methods, Fig. 5B and C). Compared to the null hypothesis of no taxa changes, both merged SC and LC showed statistically significant taxa displacement over time, even 2 months after merging with SynCom (Fig. 5B and C). As a threshold for displacement, we used a 99.7% confidence interval (i.e. ± three times the standard error), calculated from the residual variation among paired ASVs in merged vs. non-merged controls at *t* = 0 (when there is no influence of a merger). We verified this by comparing paired ASV data dispersion among non-merged community replicates alone, which was smaller than between merged-non-merged pairs (Fig. S6A–C). With outliers being defined as paired ASVs outside the calculated confidence interval, total outlier distance sums (summed distances of enriched and depleted outlier ASVs) were higher for the coalesced LC s than for the SCs (Fig. 5D), and higher than the summed random outlier distances within non-merged LC or SC (Fig. 5D). This indicated that the coalescence of SC and LC with SynComs was characterized by a higher degree of disturbance exerted by SynCom on the LC resident community.

At phylum level, coalescence resulted in exclusive depletion of *Firmicutes*-ASVs, without a single enriched *Firmicutes*-ASV (Fig. 5E). Also, *Gemmatimonadetes*-ASVs were mostly depleted upon SynCom merging (Fig. 5E), and their total abundance diminished in SC mergers (Fig. S6D). In contrast, the abundances of *Bacteroidetes*, *Proteobacteria,* and *Verrucomicrobia* phyla increased with enriched and depleted ASV outliers at all time points (Fig. 5A). Some phyla showed pronounced late responses; like the enrichment of outliers in the *Actinobacteria* and *Chloroflexi* phyla in SC, and in the *Cyanobacteria* phylum in LC (Fig. 5E).

**Figure 6:**
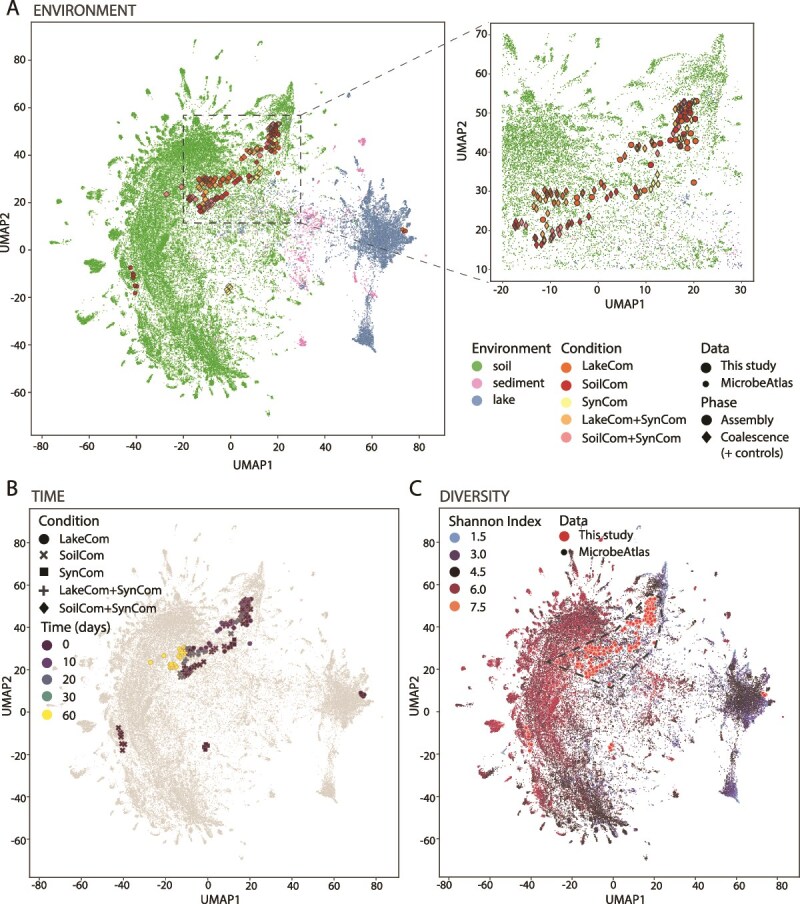
LC and SC developmental trajectories cluster with global natural soil microbiome compositions. (A) UMAP clustering of all experimental soil microcosm samples (pre- and post-coalescence), and available soil (in green), lake sediment (light pink), and lake water microbiome (blue) samples from the MicrobeAtlas database. Colors and shapes specify different environments and sample origins as per the figure legend. The subplot on the right is an expansion of the main UMAP region with shapes relating to our samples enlarged manually for clarity (indicated with the dashed lines and rectangle). (B) As in (A) but highlighting the time progression of the soil microcosm trajectories to (C) higher Shannon index. The polygon with the dotted black line was added manually for clarity and includes all experimental samples except for the inocula of SC, SynCom, and LC.

Placing all merged and non-merged community compositions in a UMAP for dimension reduction, processed at 97% OTU threshold definition with the 81 627 publicly available characterized natural soil and lake communities in MicrobeAtlas, showed that both SynCom-merged and unmerged LC and SC resemble other soil microbiomes (Fig. 6A). The soil signature drifted from the intermediary zone of the projection in the first incubation phase (Fig. S2E), to a more central position within the soil cluster of MicrobeAtlas in the second phase and in the merged communities (Fig. 6B). The central position is marked by higher diversity natural soil microbiomes and suggests maturation of the microcosm communities (Fig. 6C).

### SynCom age in soil microcosms determines its colonization resistance to SoilCom

Considering the unexpectedly poor proliferation of SynCom within the coalesced communities, we investigated the potential mechanisms underlying its decline. Assuming habitat factors play an important role here, we cultured SynComs to different stages of aging in their soil microcosms (2 weeks and 3 months), and then mixed fresh SC inoculum (at ca. 0.1% of the measured SynCom size, without further nutrients; Fig. 1B). If nutrient competition was the reason for SynCom decline, we expected soil-grown SynComs at different timepoints of incubation to show varying resistance to invasion of taxa-diverse SC.

**Figure 7:**
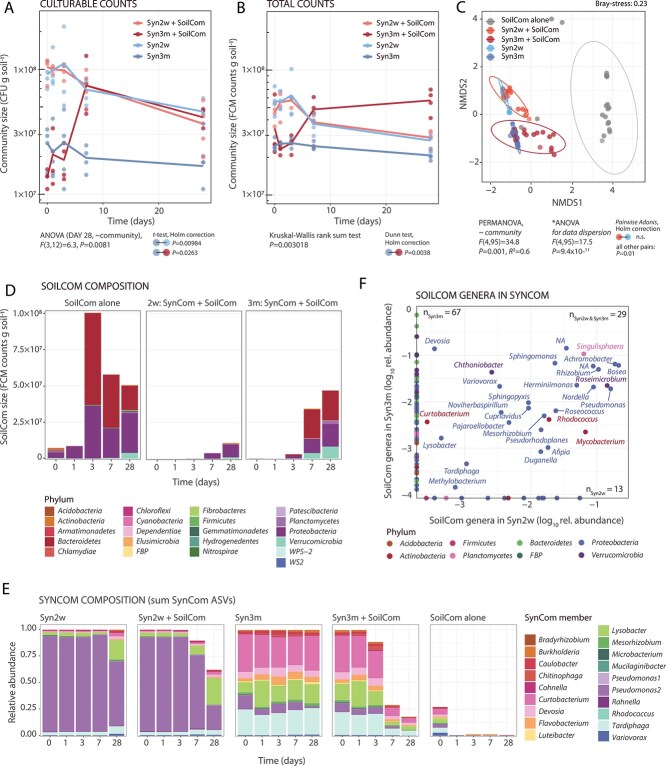
SynCom aging decreases colonization resistance to SC invasion. (A and B) Community sizes over time in merged or control communities, quantified by (A) CFU counting or (B) flow cytometry total cell counts. Circles represent individual replicate values with lines connecting their means (*n* = 4 replicates). Circle colors represent Syn2w (in light blue), Syn2w + SC (orange), Syn3m (dark blue), and Syn3m + SC (red). Differences between conditions were investigated using one-way ANOVA and post hoc *t-*tests (Holm’s correction) on the last time point CFU measurement (A), or by Kruskal–Wallis rank sum and post hoc Dunn tests (with Holm’s correction) for flow cytometry (B). Only significant *P*-values (<.05) are indicated. (C) Non-metric multidimensional scaling (NMDS) ordination based on paired ASV-level Bray–Curtis distances. Circles represent individual replicate and time point values, colored by condition (as per the color scale within the panel). Ellipses show multivariate t-distribution connection of conditions (same color code). Differences between communities were tested by PERMANOVA, and ANOVA was used to verify data dispersion. *P*-values of pairwise post hoc Adonis tests are indicated next to the panel using connected circles to indicate the comparison (Supplementary data). (D) Phyla composition attribution of SC taxa (as absolute abundances per g soil, derived from flow cytometry counts multiplied by ASV relative abundances) over time. Stacked bars show means of calculated abundances from 4 biological replicates. Phyla color code shown below the panel. (E) Relative abundances of SynCom members (colored and stacked by genus level according to legend next to the panel) across tested communities and time. Stacked bars show the means of relative abundances of four replicates. (F) Common and unique SC genera among Syn2w and Syn3m-merged communities at the last time point (day 28; as log_10_-transformed relative abundances). Circles are the means from *n* = 4 replicates, with colors indicating phylum-level affiliation as per the legend below the panel. *n*, number of SC genera detected exclusively in Syn2w (n_Syn2w_) or Syn3m (n_Syn3m_), or common to both (n_Syn2w&Syn3m_).

The community sizes of Syn2w alone and Syn2w + SC declined similarly and slowly over 1 month, both in CFUs and flow cytometry counts (Fig. 7A and B). Conversely, the sizes of the Syn3m communities mixed with SC inoculum increased by two- to fivefold in all replicates, compared to the Syn3m control (Fig. 7A and B), and with larger compositional shifts (Fig. 7C and D). The total relative abundance of SynCom membership in Syn3m decreased from 100% to ca. 18% 1 month after merging with SC inoculum, and that of Syn2w was reduced to ca. 62% (Fig. 7E). At genus level, we identified 29 genera in common between the SC expansion in Syn2w and Syn3m backgrounds, 67 genera unique for Syn3m, and 13 for Syn2w (Fig. 7F). Given that Syn3m is more aged than Syn2w, it is unlikely that invading SC members after 3 months can use nutrients that were not accessible at 2 weeks of SynCom growth. Rather, it suggests that nutrients were liberated as a consequence of 3 months of SynCom maturation, for example, in form of dead cells (the viable SynCom size after 3 months is one-third of that after 2 weeks; see Fig. 4B).

## Discussion

To counteract microbiome dysbiosis, strategies using transplantation have gained popularity [11, 32, 33]. Transplantation consists of mixing into the dysbiosed system a taxa-diverse microbiota, directly from its native habitat or in some purified preparation. Transplanting may allow recolonization of the dysbiosed habitat, reset community development, and restore a well-functioning microbiome. Although the clinical use of microbiota transplants has been successful in treating recurrent *Clostridioides difficile* infection [17, 18], the processes and key factors underlying microbiota mergers are poorly understood [34].

By culturing two distinct taxa-diverse communities (SC and LC), and a defined synthetic soil community (SynCom) under controlled conditions in pristine soil habitats, first separately and then merged, we sought to distinguish the effects of two counteracting processes on community development: (i) microbiota origin: taxa memberships and their fundamental metabolic capacities [25], relative abundances in the starting community, and inherent potential for forming interspecific interactions, and (ii) habitat filtering: the nutritional and spatial niches that select the realized metabolic capacities and interactions.

Despite different origins, both soil and lake starting communities displayed similar taxa successions, and converged to typical soil microbiota when allowed to proliferate in the same soil habitat (Fig. 2C). This indicates the dominant role of habitat to select the proliferation of microorganisms with appropriate functional traits and types of interspecific interactions, but within the limits of their seeding composition. Next, we forced new starting mixtures by merging and diluting soil-microcosm-grown communities into the same (but uncolonized) habitat. We expected that drastically changing the starting microbiota composition and relative abundances in the mergers (e.g. LC +SynCom) would lead to different succession patterns. However, merged communities and non-merged controls displayed largely similar growth trajectories (Fig. 5A), from which we conclude that differences in the starting communities (and thus their fundamental functionalities and inherent interactions) played a minor role compared to the habitat filtering process.

Our results thus present experimental support for the original hypothesis of Baas-Becking, who stated that: “Everything is everywhere, but the environment selects” [35]. Assuming that microbes are dispersed everywhere, he suggested that environmental selection drives between-community differences [35]. The premise that all (microbial) species are everywhere has been challenged by deep taxonomic sequencing of microbiota compositions in different habitats, indicating that most environments tend to have specific community signatures and dispersal is effectively limiting all microbes to be everywhere [11, 36, 37]. The colonization and selection of a soil-like community from lake water origin in our experiments likely worked, because lake inoculum already contained taxa capable of growing in the soil habitat, as a result of the natural dispersal from soil into lake via run-off waters [26, 38, 39]. Obviously, habitat selection did not produce identical communities and, although globally similar, the soil community resulting from the lake inoculum maintained a signature different from the soil-grown soil inoculum.

Taxa comparisons suggested that community differences were not caused by genera exclusive to either LC or SC inoculum, but rather by the underperformance of common genera in LC to compensate a rapid decline observed after day 3 in the soil microcosms for both communities (Fig. 3B). This may be due to strain-specific functional differences that we did not capture in our sequencing approach. LC trajectories were also characterized by more variation in the proportion of genera with increasing or declining populations compared to SC (Fig. S3C). We may assume that the soil inoculum members coevolved to some extent to use soil nutrients more optimally in a collective food web via metabolite leakage and cross-feeding, resulting in better community growth in the soil [40–43]. A high-diverse taxa inoculum (as in LC) would thus not automatically produce higher total community biomass [40, 44, 45].

Little is known about the processes underlying microbial community development and coalescence [19]. Some studies have related coalescence outcomes to the more productive source community [46], but we show here that productivity is not a general decisive factor considering that the more-productive SynCom was outcompeted in the same habitat by the less-productive LC. The 4–5 orders of magnitude decline of the merged SynCom members in both taxa-diverse backgrounds (LC and SC) over 60 days was contrary to our expectations (Fig. 4), given that SynCom members originate from soil. This may be interpreted as a sign of poor competitiveness, but is the result of the collective types of interactions emerging in the developing communities, including direct substrate competition, metabolic facilitation [47], predation, or cell death. The final relative abundances of SynCom members in the merged communities were actually similar as their natural occurrence in the starting soil (Fig. 4), suggesting that it is the number of redundant strains and the starting mixture sharing nutritional niches that controls their final relative abundances. Despite the poor ultimate survival of SynCom members and the similar growth trajectories of merged communities vs. their non-merged controls, the temporal effects of the SynCom presence were clearly quantifiable, affected the LC stronger than the SC, and influenced multiple phyla (Fig. 5).

Some differences between the mergers of LC or SC with SynCom point to predation and cell death (e.g. the steep declines from day 3–7 in Fig. 3B). Particularly, merged LC was characterized by a temporal blooming of the *Lysobacter* isolate from SynCom (Fig. 4E). Considering that *Lysobacter* is described as facultative predator [48, 49], its temporal blooming may be the result of predation on LC members, and may have contributed to the higher variability and taxa displacement observed in LC +SynCom. Unfortunately, we have no specific information on the relative abundances of potential other predators or phages in LC and SC, which makes it difficult to estimate the roles of predatory control on growth of the merged communities as a whole, or of specific populations present in either of the starting communities. The role of cell death and viability is also evident from the invasion, proliferation, and eventual dominance of SC inoculum into pre-colonized SynComs (Fig. 7D). Coalescence outcomes thus depend on functional overlaps and differences among individual taxa, their interactions during community development as a function of the dynamic nutritional niche availabilities, predation, and the proportion of viable cells [50].

Our results are significant for understanding the behavior of coalescing communities and set a basis for developing transplants as means to restore dysbiosed soil communities. The consistent SynCom decline is reason for caution when considering coalescence with synthetic guilds for precise microbiome interventions [23, 51]. Still, even its transient presence was sufficient to consistently and profoundly change the trajectories of the merged communities (Fig. 5A and E). This is in agreement with previous studies that found permanent changes in defined synthetic communities even after transient inoculant survival [52, 53]. For soil microbiome restorations, it may thus not be sufficient to choose transplant-taxa on the basis of their origin and assume they will proliferate and integrate into the target community (at high levels). The outcomes of the coalescence rather depend on emerging community interaction networks and functional niche redundancies, the influence of which at this point is hard to predict. However, the strong environmental filtering by the soil habitat as observed here can steer transplanted merged microbiomes in the direction of natural soil microbiome types. In conclusion, our study presents a new approach for the de novo generation of soil-like microbiomes and mergers, offering new perspectives for interventions in soil restoration management.

## Supplementary Material

Causevic_Supp_methods_figs_data_ref_wraf162

## Data Availability

Community profiling data (V3–V4 16S rRNA gene amplicon sequences) is accessible through BioProject ID PRJNA1192999. All code for data processing and raw data, organized per manuscript figure are available for download from Zenodo [54].
