# Supplementary material for: Habitat filtering more than microbiota origin controls microbiome transplant outcomes in soil
Source: ISME J. 2025 Aug 1;19(1):wraf162. doi: 10.1093/ismejo/wraf162 (PMC12368958; doi:10.1093/ismejo/wraf162)
Supplement: Causevic_Supp_methods_figs_data_ref_wraf162 [file causevic_supp_methods_figs_data_ref_wraf162.docx]

Supplementary Materials for

**Habitat filtering more than microbiota origin controls microbiome transplant outcomes in soil**

Causevic *et al.*

*Corresponding author. Email: [senka.causevic@unil.ch](mailto:senka.causevic@unil.ch)

**This file includes:**

Supplementary methods

Figs. S1to S6

Supplementary data

Supplementary references

**Supplementary methods**

**Soil buffer composition**

Soil buffer contained per L, 0.6 g of MgSO_4_·7H_2_O, 0.1 g of CaCl_2,_ and 1.8 mL of 5 × M9 minimal salts solution (BD Biosciences).

**SoilCom preparation**

Previously described in [1]. Large debris (e.g. rocks, branches, and leaves) were manually removed, soil was immediately homogenized, and sieved through a 2 mm-grid size sieve. 250 g of sieved soil was mixed with 500 ml of tetrasodium-pyrophosphate decahydrate solution (Sigma-Aldrich, 0.2% wt/vol, pH 7.5), homogenized for 2 min at 2000 rpm in a Stare SM4 blender (Satrap), and left idle to sediment for 30 min. The supernatant was divided to 50 ml polypropylene centrifuge tubes and centrifuged at 800 rpm for 5 min in an Eppendorf A-4-62 Swing Bucket Rotor to remove large particles. The upper phase was collected and layered over 10 ml sterile 60% w/v Histodenz solution in water (Sigma-Aldrich, D2158, density of 1.31 g ml^-1^) in 50 ml tubes, which were centrifuged at 3220 × g for 30 min at room temperature (Eppendorf A-4-62 Swing Bucket Rotor). The interface layer containing microbial cells was recovered, diluted five times with pyrophosphate solution, and subsequently centrifuged at 3220 × g for 10 min as above. The resulting cell pellet was resuspended in 5 ml of soil buffer, and an aliquot was taken for cell counting by flow cytometry.

**LakeCom preparation**

Water was sampled (in February 2023) ca. 5 m away from the quay wall with a sterile stainless-steel bucket and immediately transported to the laboratory. Microbial biomass was collected from 10 litres of water filtered through five 47 mm diameter 0.2–µm polyethersulfone membrane filters (Type 15407, Sartorius Stedim Biotech) using a sterile glass filter holder (Millipore). Microbial cells were detached from each filter by 1 min vortexing in 5 ml sterile lake water and pooled together. Sterile lake water was prepared in advance using the same sampling and filtering procedure, but keeping the filtrate, which was subsequently autoclaved for 30 min at 121°C.

**Soil microcosm preparation**

Previously described in [1]. The dried riverbank sediment was sieved to obtain a particle fraction ranging between ca. 0.5 and 3 mm, divided into 2 kg portions, and autoclaved twice for 1 h at 121°C (followed by a dry cycle), with one week storage at 23°C in between. Aliquots of 90 g of the autoclaved matrix were then transferred into 500-ml capped Schott glass bottles and autoclaved once more. To prepare soil extract (SE), freshly collected forest topsoil was mixed with tap water in a 1:1 *w/v* ratio in 5 L stainless steel containers, autoclaved for 1 h at 121°C, mixed, and left to sediment overnight. Subsequently, the supernatant was decanted, centrifuged at 5000 × g for 15 min to remove insoluble solids, transferred to 500-ml glass bottles, and autoclaved again. Both soil matrix and SE were checked for sterility by plating on R2A agar, and were considered sterile if no colonies were detected after one week incubation at 23 °C.

**Effect of aging on coalescence**

To test aging effects on coalescence, SynComs were grown for two weeks (Syn2w) or three months (Syn3m), after which ca. 1 ml of fresh SoilCom inoculum (for preparation, see above, but diluted in soil buffer to avoid adding extra carbon) was added to achieve 10^5^ cells g^–1^ microcosm material (Fig. 1b). No further SE-solution was added. As control, the same density of SoilCom inoculum was inoculated in new soil microcosms comprised of sterile matrix and deionised water (without SE). Merged and control microcosms were sampled immediately after inoculation and mixing, and then further after 1, 3, 7, and 28 days of incubation.

**MicrobeAtlas analysis**

MicrobeAtlas was filtered for soil, lake, and sediment samples as follows. All samples with extracted meta-data annotations ‘soil’, ‘soil|plant’ (main environment), or ‘aquatic;lake’, ‘aquatic;lake|sediment’ (main + sub environment) were kept, with ‘soil|plant’ typically corresponding to rhizosphere samples according to manual checks, resulting in 184,448 samples. Samples with sub-environment ‘lake|sediment’ were labeled as ‘sediment’ for downstream analyses. To further exclude potential mislabelled samples, we employed a conservative nearest-neighbour-based approach where only samples whose closest neighbouring samples predominantly shared the same main environment annotation as the focal sample were retained (one neighbouring sample per project, up to 10 projects, min. Bray-Curtis similarity 0.2). Although this excluded many potentially relevant samples, it helped ensure consistency and reduce noise of our sample sets. For ‘lake|sediment’ samples, both ‘soil’ and ‘aquatic’ neighbours were considered matches. We manually checked the ‘organism’ annotation field of all remaining 119,511 samples and blacklisted problematic terms such as ‘phyllosphere metagenome’, ‘seawater metagenome’, and ‘uncultured eukaryote’, as well as food- or host-related terms, which resulted in 109,503 samples. We then removed samples that contained more than 10% eukaryotic reads to conservatively remove samples targeting the eukaryotic fraction, leaving 81,627 total samples.

**Data analysis (full version)**

Data processing and statistical analysis were done using MATLAB (v.2021b) and R 4.0 (R Core Team, 2019) on RStudio (version 2022.2.3.492) using the following packages: *phyloseq* [2], *microbiome* [3], *vegan* [4], *biomformat* [5], *tidyverse* [6], *dplyr* [7], *reshape2* [8], *robCompositions* [9], *PMCMRplus* [10], *pairwiseAdonis* [11], *rstatix* [12], *emmeans* [13], *MicrobiotaProcess* [14], *ggplot2* [15], *ggpubr* [16], *Biostrings* [17], *DescTools* [18], *car* [19], *multcomp* [20], and *RVAideMemoire* [21].

Flow cytometry data were imported with the *fca_readfcs* [22] function on MATLAB and analysed using custom scripts (gating procedure shown in Fig. S1).

Data normality and homogeneity of variance were always checked (where applicable) using Shapiro-Wilk’s normality test and Levene’s test, respectively. Differences in community sizes of LakeCom and SoilCom during assembly and after coalescence with SynCom, differences in LakeCom and SoilCom diversity values over time, and SynCom absolute abundances within different conditions (across time) were compared using two-way repeated measures ANOVA (on ranked values when data was non-normal, otherwise on measured values), checking for the effect of community and incubation time. The presence of outliers was tested using the *identify_outliers* function (*rstatix* package). Sphericity of data was checked automatically (as a part of *anova_test* function, *rstatix* package) and corrected using Greenhouse-Geisser correction. Residuals were checked for normality and homogeneity of variance as indicated above. *Post hoc* pairwise testing was done using *t*-tests and *P* values were always adjusted using Holm’s method. Community development (with LakeCom and SoilCom assembly, and upon their coalescence with SynCom), was compared by sample ordination using NMDS on Bray-Curtis pairwise distances at ASV, Genus, or Family levels (level is always indicated on the figure). In all cases, PERMANOVA (with 999 iterations, performed using *adoniS3* function) was used to evaluate the influence of time and community on measured distances. Data homogeneity was checked with *betadisper* (*vegan* package). Pairwise differences between samples were then checked using the *pairwise.adonis* function from the *pairwiseAdonis* package (*P* values adjusted with Holm’s method).

Absolute taxa abundances were calculated by multiplying the total community size (measured with flow cytometry) with the relative abundance of that taxon obtained with amplicon sequencing in the same sample (see above). Community demography in SoilCom and LakeCom in Phase I and Phase II was then estimated from the per-genus individual absolute abundance differences between day 1–3, day 3–7, or day 7–21. Taxa were then grouped by being shared to both community types or exclusive to either, and further by increasing or decreasing in both, or showing opposite behaviour. The summed contribution of the taxa groups to overall community growth or decline was then quantified from the total taxa abundances at each time point.

ASV-level shifts due to mergers with SynCom were evaluated by pairing abundances of common ASVs found in the following comparisons: LakeCom+SynCom vs. LakeCom, SoilCom+SynCom vs. SoilCom, and LakeCom or SoilCom replicates by themselves in all possible paired combinations. The last two comparisons were used to evaluate baseline replicate-level variation in the data. SynCom ASVs were removed, and all samples were subsampled randomly to a density of 100,000 reads. Within each comparison group, ASVs were paired (e.g. value X = abundance in the non-merged community, value Y = same ASV in the merged community) and abundances were log_10_ transformed. A linear regression was calculated on the T_0_ data per comparison group, to obtain slope coefficients and outlier thresholds (defined as 99.7% confidence interval, ± 3 standard error of residual distribution). The T_0_ slope and thresholds were then imposed on the data from the remaining time points to detect outlier ASVs surpassing the threshold. The total outlier distance was then obtained by summing the distance of each outlier ASV to its expected value (per condition, time point, and optionally per direction - enriched or depleted). Total outlier distances were compared across all time points, and for all four comparison groups in a repeated measures ANOVA on ranked values (as described above). To further inspect the effect of SynCom transplants, the distribution of residuals from a comparison of non-merged vs. merged communities was compared to the residuals from the paired comparison of either LakeCom or SoilCom replicates alone (randomly subsampled to obtain equally sized data sets). In that case, the difference between conditions was tested as a difference in data dispersion using Levene’s test for homogeneity of variance (*P* values adjusted using Holm’s method, Fig. S6a). In addition, the median values were compared with a Wilcoxon rank sum test (shown in Supplementary data). Source tracking analysis was conducted using the FEAST R package (version 0.1.0, EM_iterations = 10,000, minimum OTU prevalence: 10%) [23].

For the SynCom reverse transplant experiments with SoilCom inoculum, the CFU count differences between conditions (Syn2w, Syn2w+SoilCom, Syn3m, Syn3m+SoilCom) were tested at the last time point with a one-way ANOVA (assumptions and residuals checked as before) and *post hoc t*-tests (Holm’s correction). The last time point comparison using flow cytometry data and for Shannon Index was made in a Kruskal-Wallis rank sum test and *post hoc* Dunn test with Holm’s correction. Differences in beta diversity of each condition (Bray-Curtis distances) were checked as described above.

UMAP [24] projections were generated from Bray-Curtis distance matrices (computed on relative abundances, normalized including unmapped reads) using julia 1.9.3 [25] and the *Distances.jl* package [26] (version 0.10.11). UMAP projections were computed using the python *umap* package [27] (version 0.5.3; parameters: n_neighbors = 3000, min_dist = 1.5, spread = 15, epochs = 500). Scatterplots were produced using python 3.9.9 [28] and the *seaborn* package [29] (version 0.13.2). Outlier studies detected in the UMAP projection were labelled using the *dbscan* algorithm from the *Clustering.jl* package [30](version 0.15.2; parameters: radius = 1.5, min_cluster_size = 5), leaving 63,750 samples that underwent a final round of UMAP projection with identical parameters. Samples from this study were finally mapped onto this UMAP projection using the transform() function of the *umap* object. Shannon Index for each sample was computed using the *Microbiome.jl* package [31](version 0.10.1).

For the differential abundance analysis in Fig. S2e, a distinct, intermediary zone (IZ) of the projection was delineated based on i) the initial trajectory of SoilCom and LakeCom samples, and ii) cluster distinctness. This resulted in 3733 IZ and 60,017 non-IZ samples. To account for sample imbalance and computational complexity, 1000 samples of each group were randomly selected for further analysis. To check for differentially abundant 97% OTUs between the two zones (IZ and non-IZ), we used SiamCat v.2.2.0 [32] in R 3.6.3. All OTUs with a prevalence of <0.5% or a maximum abundance of <0.0001% were excluded. The associations were tested with a Wilcoxon Rank-sum test, adjusted for multiple testing via the Benjamini Hochberg method. *P*=0.05 was used as our significance threshold. We then created a *siamcat* object to plot the results in an association plot, and to assess fold change of OTUs in IZ versus non-IZ.

**Supplementary figures**

**Fig. S1. Gating strategy for community size measurement using flow cytometry data. (a)** Flow cytometry events of not inoculated microcosm example (negative control) stained with Sybr Green II. From left to right, events in FSC-H vs. SSC-H, FSC-H vs. FITC-H, and SSC-H vs. FITC-H. Colour scale from blue to bright yellow (white) relates to event density. Community gate established from the last comparison as indicated on the figure. **(b)** As in (a), but for non-stained LakeCom+SynCom sample from day 3 measurement. **(c)** As in (a) and (b), but for stained LakeCom+SynCom sample.

**Fig. S2. Changes in diversity of soil and freshwater communities in same habitat. (a, b)** Community development over time in the soil microcosms of SoilCom (in green) and LakeCom (in blue), estimated on washed cell suspensions by viable counts (a, colony forming unit, CFU, counts per gram of soil), or genome equivalents (calculated from quantified DNA concentrations, b). Lines connect the means of the four biological replicates per condition (presented as dots). *P* values relate to the effect of community type on attained population sizes, as obtained with two-way repeated measures ANOVA on ranked values (Supplementary data). **(c)** Shannon index values (individual dots) describe diversity of SoilCom and LakeCom biological replicates (4 per condition) over time. Colour gradient follows the progression of time (from grey to purple), and black lines indicate mean per condition and time point. Two-way repeated measures ANOVA on ranked values was used to evaluate effect of community type and time on measured values. **(d)** NMDS ordination based on Bray-Curtis pairwise sample distances calculated from family-level compositions. Colour code as in (a), circles and diamonds depict SoilCom and LakeCom condition, respectively. PERMANOVA with 999 iterations was used to evaluate the effect of community and time (see Materials and Methods and Supplementary Data). **(e)** UMAP projection of SoilCom, LakeCom, and MicrobeAtlas samples, coloured by IZ (intermediary zone, red) and non-IZ (blue) regions. On the side panel, the abundance of top 13 significantly enriched OTUs in IZ zone is plotted (comparison of relative abundances of OTUs in 1000 randomly selected IZ (red) and non-IZ (blue) samples).

**Fig. S3. SoilCom and LakeCom compositional trajectories. (a)** Values (presented as dots, 4 biological replicates) obtained using relative abundance of each phylum and total community size measured with flow cytometry. Lines connect mean values per condition over time. Colour code as indicated on the figure. Phyla only showing a quick decline from day 0 to 1 are not included. **(b)** Log_2_-fold-change in absolute genera abundances (summed from their individual ASVs) in LakeCom versus SoilCom during the indicated time intervals (e.g., Day 1 – Day 3), highlighting similar behaving groups (e.g., increase in both LakeCom and SoilCom). Circles display the means of log_2_-fold-changes from four replicates, which are coloured by phylum according to the legend in panel (c). **(c)** Count of genera (coloured by phyla affiliation) which declined or increased their absolute abundance according to indicated time interval in LakeCom and/or SoilCom. See figure 3b.

**Fig. S4. SynCom compositional dynamics during Phase I. (a)** Stacked barplot depicts contribution of each of the 21 members of SynCom community to its dynamics starting from the first day after microcosm inoculation to 21^st^ day. In this experiment 4 biological replicates were followed.

**Fig. S5. Outcomes of microbiome coalescence according to FEAST analysis. (a)** Estimated proportions of source communities (LakeCom, SoilCom, and SynCom) within merged LakeCom+SynCom communities over time, as predicted by the microbial source tracking algorithm FEAST. SoilCom was included as a control. **(b)** Same as (a), but for merged SoilCom+SynCom communities. LakeCom was included as a control.

**Fig. S6. Baseline SoilCom and LakeCom taxa displacement over time. (a)** Histograms show residual distribution over time for the merged vs. non-merged community comparison (dark colours), and the corresponding non-merged community by itself (random replicate pairs; white colour). *P* and *F* values indicate distribution comparisons using Levene’s test for homogeneity of variance. **(b)** Dots present ASV abundances paired within replicates (all possible combinations) of non-merged SoilCom (previously subsampled to 100000 reads) over time (time points presented as shades of green). Black line shows the slope obtained on T_0_ data comparison, which is used to define outliers of all time points (based on a 99.7% confidence interval). Outliers are presented as dots with magenta circles. **(c)** As in (b), but for comparison among non-merged LakeCom replicates. **(d)** Absolute phyla abundances of the non-SynCom fraction per community type over time (calculated as the remaining proportion from (4e), multiplied by the total community size as measured with flow cytometry). Phyla colours indicated on the right of the panel.

**Supplementary data**

**Table S1. SynCom composition.** SynCom consists of 21 soil isolates obtained from forest topsoil (same location as SoilCom inoculum). It was previously described in [1].

| No. | Genus |
| --- | --- |
| 1 | *Variovorax* |
| 2 | *Flavobacterium* |
| 3 | *Microbacterium* |
| 4 | *Mucilaginibacter* |
| 5 | *Curtobacterium* |
| 6 | *Rhodococcus* |
| 7 | *Bradyrhizobium* |
| 8 | *Cellulomonas* |
| 9 | *Luteibacter* |
| 10 | *Phenylobacterium* |
| 11 | *Mesorhizobium* |
| 12 | *Lysobacter* |
| 13 | *Pseudomonas* |
| 14 | *Caulobacter* |
| 15 | *Pseudomonas* |
| 16 | *Devosia* |
| 17 | *Cohnella* |
| 18 | *Tardiphaga* |
| 19 | *Burkholderia* |
| 20 | *Rahnella* |
| 21 | *Chitinophaga* |

Supplementary figure 2A statistics

# *Day 0 removed from analysis.*

# Shapiro-Wilk normality test soil

| W = 0.89525 | p-value = 0.06748 |
| --- | --- |

# Shapiro-Wilk normality test lake

| W = 0.91394 | p-value = 0.1347 |
| --- | --- |

# Levene's Test for Homogeneity of Variance (center = median)

| Df | F value | Pr(>F) |
| --- | --- | --- |
| 7 | 3.7403 | 0.007072 |
| 24 |  |  |

# Outliers

| Condition | Time | Sample | Phase | CFU | ranked value | is.outlier | is.extreme |
| --- | --- | --- | --- | --- | --- | --- | --- |
| LakeCom | 21d | LW-2 | 1 | 4400000 | 9 | TRUE | FALSE |
| LakeCom | 7d | LW-3 | 1 | 19000000 | 20 | TRUE | FALSE |
| SoilCom | 21d | NC2-4 | 1 | 90800000 | 24 | TRUE | FALSE |

# Repeated measures two-way ANOVA with ranked values (otherwise residuals are non-normal)

| Effect | DFn | DFd | F | p | ges |
| --- | --- | --- | --- | --- | --- |
| Condition | 1 | 6 | 351.086 | 1.49E-06 | 0.902 |
| Time | 3 | 18 | 225.716 | 1.82E-14 | 0.969 |
| Condition:Time | 3 | 18 | 49.058 | 7.25E-09 | 0.873 |

# Pairwise *t*-test (on ranked values), Holm’s correction

| Time | .y. | group1 | group2 | n1 | n2 | p | p.adj | p.adj.signif |
| --- | --- | --- | --- | --- | --- | --- | --- | --- |
| 1 | ranked value | LakeCom | SoilCom | 4 | 4 | 0.00412 | 0.00412 | ** |
| 3 | ranked value | LakeCom | SoilCom | 4 | 4 | 0.0000392 | 0.0000392 | **** |
| 7 | ranked value | LakeCom | SoilCom | 4 | 4 | 0.0000895 | 0.0000895 | **** |
| 21 | ranked value | LakeCom | SoilCom | 4 | 4 | 0.0000176 | 0.0000176 | **** |

# Residuals

# Shapiro-Wilk normality test

| W=0.95665 | p-value=0.222 |
| --- | --- |

# Levene's Test for Homogeneity of Variance (center = median)

| Df | F value | Pr(>F) |
| --- | --- | --- |
| 7 | 0.3624 | 0.9151 |
| 24 |  |  |

Figure 2A statistics

# *Day 0 removed from analysis.*

# Shapiro-Wilk normality test soil

| W = 0.8788 | p-value = 0.01684 |
| --- | --- |

# Shapiro-Wilk normality test lake

| W = 0.72526 | p-value = 8.056E-05 |
| --- | --- |

# Levene's Test for Homogeneity of Variance (center = median)

| Df | F value | Pr(>F) |
| --- | --- | --- |
| 9 | 4.7499 | 0.0005627 |
| 30 |  |  |

# Outliers

| Community | Time | Sample | Phase | Per g cell | is.outlier | is.extreme |
| --- | --- | --- | --- | --- | --- | --- |
| SoilCom | 3d | NC2-4 3day | 1 | 120875480 | TRUE | FALSE |

# Repeated measures ANOVA with ranked values

| Effect | DFn | DFd | F | p | ges |
| --- | --- | --- | --- | --- | --- |
| Community | 1 | 6 | 130.39 | 2.71E-05 | 0.85 |
| Time | 4 | 24 | 179.376 | 1.67E-17 | 0.957 |
| Condition:Time | 4 | 24 | 17.362 | 8.17E-07 | 0.681 |

# Pairwise *t*-test (on ranked values), Holm’s correction

| Time | .y. | group1 | group2 | n1 | n2 | p | p.adj | p.adj.signif |
| --- | --- | --- | --- | --- | --- | --- | --- | --- |
| 1 | ranked value | LakeCom | SoilCom | 4 | 4 | 0.429 | 0.429 | ns |
| 3 | ranked value | LakeCom | SoilCom | 4 | 4 | 0.00208 | 0.00208 | ** |
| 7 | ranked value | LakeCom | SoilCom | 4 | 4 | 0.0000525 | 0.0000525 | **** |
| 10 | ranked value | LakeCom | SoilCom | 4 | 4 | 0.000109 | 0.000109 | *** |

# Residuals

# Shapiro-Wilk normality test

| W=0.96666 | p-value=0.2807 |
| --- | --- |

# Levene's Test for Homogeneity of Variance (center = median)

| Df | F value | Pr(>F) |
| --- | --- | --- |
| 9 | 0.5018 | 0.8614 |
| 30 |  |  |

Supplementary figure 2B statistics

# *Day 0 and 7 removed from analysis.*

# Shapiro-Wilk normality test soil

| W = 0.92635 | p-value = 0.2133 |
| --- | --- |

# Shapiro-Wilk normality test lake

| W = 0.76533 | p-value = 0.000978 |
| --- | --- |

# Levene's Test for Homogeneity of Variance (center = median)

| Df | F value | Pr(>F) |
| --- | --- | --- |
| 7 | 3.5784 | 0.008889 |
| 24 |  |  |

# Outliers: no

# Repeated measures ANOVA with ranked values

| Effect | DFn | DFd | F | p | ges |
| --- | --- | --- | --- | --- | --- |
| Condition | 1 | 6 | 8.701 | 2.60E-02 | 0.294 |
| Time | 3 | 18 | 143.470 | 9.36E-13 | 0.945 |
| Condition:Time | 3 | 18 | 9.866 | 4.53E-04 | 0.540 |

# Pairwise *t*-test (on ranked values), Holm’s correction

| Time | .y. | group1 | group2 | n1 | n2 | p | p.adj | p.adj.signif |
| --- | --- | --- | --- | --- | --- | --- | --- | --- |
| 1 | ranked value | LakeCom | SoilCom | 4 | 4 | 0.897 | 0.897 | ns |
| 3 | ranked value | LakeCom | SoilCom | 4 | 4 | 0.0274 | 0.0274 | * |
| 14 | ranked value | LakeCom | SoilCom | 4 | 4 | 0.00862 | 0.00862 | ** |
| 21 | ranked value | LakeCom | SoilCom | 4 | 4 | 0.00862 | 0.00862 | ** |

# Residuals

# Shapiro-Wilk normality test

| W= 0.97222 | p-value= 0.5628 |
| --- | --- |

# Levene's Test for Homogeneity of Variance (center = median)

| Df | F value | Pr(>F) |
| --- | --- | --- |
| 7 | 1.0455 | 0.4269 |
| 24 |  |  |

Supplementary figure 2C statistics

# Shapiro-Wilk normality test soil

| W= 0.92227 | p-value= 0.07453 |
| --- | --- |

# Shapiro-Wilk normality test lake

| W= 0.86056 | p-value= 0.003455 |
| --- | --- |

# Levene's Test for Homogeneity of Variance (center = median)

| Df | F value | Pr(>F) |
| --- | --- | --- |
| 11 | 1.8686 | 0.07901 |
| 35 |  |  |

# Outliers

| Condition | Time | Sample | Phase | Shannon | is.outlier | is.extreme |
| --- | --- | --- | --- | --- | --- | --- |
| LakeCom | 0 | LW1 t0 | 1 | 5.01 | TRUE | FALSE |
| LakeCom | 7 | LW1 d7 | 1 | 5.25 | TRUE | FALSE |
| SoilCom | 3 | NC2 2 d3 | 1 | 4.11 | TRUE | FALSE |

# Repeated measures ANOVA on ranked values

| Effect | DFn | DFd | F | p | ges |
| --- | --- | --- | --- | --- | --- |
| Community | 1 | 5 | 0.613 | 4.69E-01 | 0.023 |
| Time | 5 | 25 | 21.784 | 2.23E-08 | 0.778 |
| Community:Time | 5 | 25 | 22.982 | 1.31E-08 | 0.787 |

# Pairwise *t*-test (on ranked values), Holm’s correction

| Time | .y. | group1 | group2 | n1 | n2 | p | p.signif | p.adj | p.adj.signif |
| --- | --- | --- | --- | --- | --- | --- | --- | --- | --- |
| 0 | Shannon | LA | NC | 4 | 4 | 0.00000214 | **** | 0.00000214 | **** |
| 1 | Shannon | LA | NC | 4 | 4 | 0.0169 | * | 0.0169 | * |
| 3 | Shannon | LA | NC | 4 | 4 | 0.00222 | ** | 0.00222 | ** |
| 7 | Shannon | LA | NC | 4 | 3 | 0.0000994 | **** | 0.0000994 | **** |
| 14 | Shannon | LA | NC | 4 | 4 | 0.000611 | *** | 0.000611 | *** |
| 21 | Shannon | LA | NC | 4 | 4 | 0.0002 | *** | 0.0002 | *** |

# Residuals

# Shapiro-Wilk normality test

| W= 0.83123 | p-value= 2.204e-05 |
| --- | --- |

# Levene's Test for Homogeneity of Variance (center = median)

| Df | F value | Pr(>F) |
| --- | --- | --- |
| 11 | 0.6414 | 0.7793 |
| 30 |  |  |

Figure 2B statistics

Bray-stress= 0.1050865

# Permutation test for adonis under reduced model

# Number of permutation: 999

|  | Df | SumOfSqs | R2 | F | Pr(>F) |
| --- | --- | --- | --- | --- | --- |
| group | 1 | 3.7826 | 0.21484 | 18.8865 | 0.001 |
| time | 5 | 5.8124 | 0.33014 | 5.8043 | 0.001 |
| Residual | 40 | 8.0112 | 0.45502 |  |  |
| Total | 46 | 17.6063 | 1 |  |  |

# ANOVA on dispersion

|  | Df | Sum Sq | Mean Sq | F | Pr(>F) |
| --- | --- | --- | --- | --- | --- |
| Groups | 11 | 0.39524 | 0.035931 | 3.7013 | 0.001494 |
| Residuals | 35 | 0.33977 | 0.009708 |  |  |

# Pairwise Adonis

|  | pairs | Df | SumsOfSqs | F.Model | R2 | p.value | p.adjusted | sig |
| --- | --- | --- | --- | --- | --- | --- | --- | --- |
| 1 | lake 1 | vs | lake 14 | 1 | 0.91846894 | 6.199349 | 0.5081705 | 0.031 |
| 2 | lake 1 | vs | lake 21 | 1 | 1.05261242 | 7.095662 | 0.541833 | 0.046 |
| 3 | lake 1 | vs | lake 3 | 1 | 0.34994672 | 3.48112 | 0.3671634 | 0.04 |
| 4 | lake 1 | vs | lake 7 | 1 | 0.67975248 | 4.835712 | 0.4462754 | 0.031 |
| 5 | lake 1 | vs | lake 0 | 1 | 1.62725461 | 17.083929 | 0.7400789 | 0.018 |
| 6 | lake 1 | vs | soil 1 | 1 | 1.16676133 | 5.594133 | 0.4824969 | 0.028 |
| 7 | lake 1 | vs | soil 14 | 1 | 1.39763665 | 9.754961 | 0.6191676 | 0.028 |
| 8 | lake 1 | vs | soil 21 | 1 | 1.42381787 | 10.160704 | 0.628729 | 0.027 |
| 9 | lake 1 | vs | soil 3 | 1 | 1.50898304 | 14.337452 | 0.7049778 | 0.016 |
| 10 | lake 1 | vs | soil 7 | 1 | 1.28327097 | 10.500425 | 0.6774282 | 0.027 |
| 11 | lake 1 | vs | soil 0 | 1 | 1.64154815 | 14.251112 | 0.70372 | 0.031 |
| 12 | lake 14 | vs | lake 21 | 1 | 0.17189112 | 1.464494 | 0.1961947 | 0.183 |
| 13 | lake 14 | vs | lake 3 | 1 | 1.15154982 | 16.556318 | 0.7339991 | 0.024 |
| 14 | lake 14 | vs | lake 7 | 1 | 0.35072547 | 3.200175 | 0.3478385 | 0.025 |
| 15 | lake 14 | vs | lake 0 | 1 | 1.7871038 | 27.803142 | 0.8225017 | 0.038 |
| 16 | lake 14 | vs | soil 1 | 1 | 1.25916229 | 7.090069 | 0.5416373 | 0.035 |
| 17 | lake 14 | vs | soil 14 | 1 | 1.27645875 | 11.366417 | 0.6545056 | 0.023 |
| 18 | lake 14 | vs | soil 21 | 1 | 1.26715914 | 11.608668 | 0.6592587 | 0.032 |
| 19 | lake 14 | vs | soil 3 | 1 | 1.62046502 | 21.817368 | 0.7843074 | 0.025 |
| 20 | lake 14 | vs | soil 7 | 1 | 1.31104312 | 15.416226 | 0.7550968 | 0.029 |
| 21 | lake 14 | vs | soil 0 | 1 | 1.71962965 | 20.419805 | 0.7728976 | 0.024 |
| 22 | lake 21 | vs | lake 3 | 1 | 1.35378409 | 19.410849 | 0.7638804 | 0.028 |
| 23 | lake 21 | vs | lake 7 | 1 | 0.58558807 | 5.33391 | 0.4706152 | 0.028 |
| 24 | lake 21 | vs | lake 0 | 1 | 1.78882169 | 27.747766 | 0.8222105 | 0.037 |
| 25 | lake 21 | vs | soil 1 | 1 | 1.28689141 | 7.238454 | 0.5467749 | 0.026 |
| 26 | lake 21 | vs | soil 14 | 1 | 1.35107618 | 12.010519 | 0.6668613 | 0.03 |
| 27 | lake 21 | vs | soil 21 | 1 | 1.3324075 | 12.185189 | 0.6700612 | 0.034 |
| 28 | lake 21 | vs | soil 3 | 1 | 1.6515716 | 22.179383 | 0.7870784 | 0.024 |
| 29 | lake 21 | vs | soil 7 | 1 | 1.35739903 | 15.918594 | 0.7609782 | 0.02 |
| 30 | lake 21 | vs | soil 0 | 1 | 1.71412834 | 20.308615 | 0.7719378 | 0.035 |
| 31 | lake 3 | vs | lake 7 | 1 | 0.86930273 | 14.028468 | 0.7004264 | 0.03 |
| 32 | lake 3 | vs | lake 0 | 1 | 1.92061157 | 115.363237 | 0.9505616 | 0.028 |
| 33 | lake 3 | vs | soil 1 | 1 | 1.42009443 | 10.926616 | 0.6455287 | 0.023 |
| 34 | lake 3 | vs | soil 14 | 1 | 1.60850994 | 24.871727 | 0.8056474 | 0.029 |
| 35 | lake 3 | vs | soil 21 | 1 | 1.6410149 | 26.671187 | 0.8163519 | 0.035 |
| 36 | lake 3 | vs | soil 3 | 1 | 1.61257403 | 60.51974 | 0.9098012 | 0.038 |
| 37 | lake 3 | vs | soil 7 | 1 | 1.37738112 | 49.38855 | 0.9080689 | 0.037 |
| 38 | lake 3 | vs | soil 0 | 1 | 1.87468727 | 51.241749 | 0.8951814 | 0.03 |
| 39 | lake 7 | vs | lake 0 | 1 | 1.80281369 | 31.800929 | 0.8412737 | 0.043 |
| 40 | lake 7 | vs | soil 1 | 1 | 1.25129139 | 7.360159 | 0.5509035 | 0.031 |
| 41 | lake 7 | vs | soil 14 | 1 | 1.21980364 | 11.648858 | 0.6600347 | 0.028 |
| 42 | lake 7 | vs | soil 21 | 1 | 1.24111163 | 12.219293 | 0.6706788 | 0.033 |
| 43 | lake 7 | vs | soil 3 | 1 | 1.58239356 | 23.72844 | 0.7981731 | 0.028 |
| 44 | lake 7 | vs | soil 7 | 1 | 1.2045885 | 15.862512 | 0.7603357 | 0.026 |
| 45 | lake 7 | vs | soil 0 | 1 | 1.74489289 | 22.771145 | 0.7914577 | 0.024 |
| 46 | lake 0 | vs | soil 1 | 1 | 1.61813152 | 12.977226 | 0.6838316 | 0.024 |
| 47 | lake 0 | vs | soil 14 | 1 | 1.82103108 | 30.659269 | 0.8363306 | 0.029 |
| 48 | lake 0 | vs | soil 21 | 1 | 1.83050977 | 32.541714 | 0.8443245 | 0.024 |
| 49 | lake 0 | vs | soil 3 | 1 | 1.93554034 | 90.57714 | 0.9378735 | 0.032 |
| 50 | lake 0 | vs | soil 7 | 1 | 1.65715568 | 76.873447 | 0.9389301 | 0.033 |
| 51 | lake 0 | vs | soil 0 | 1 | 1.89762853 | 60.610257 | 0.9099238 | 0.031 |
| 52 | soil 1 | vs | soil 14 | 1 | 0.89968898 | 5.209128 | 0.464722 | 0.033 |
| 53 | soil 1 | vs | soil 21 | 1 | 1.03454705 | 6.101027 | 0.5041743 | 0.034 |
| 54 | soil 1 | vs | soil 3 | 1 | 0.7043301 | 5.229379 | 0.4656873 | 0.028 |
| 55 | soil 1 | vs | soil 7 | 1 | 0.62797162 | 3.986141 | 0.4435877 | 0.058 |
| 56 | soil 1 | vs | soil 0 | 1 | 1.03380381 | 7.148076 | 0.5436595 | 0.026 |
| 57 | soil 14 | vs | soil 21 | 1 | 0.08188759 | 0.785304 | 0.115736 | 0.713 |
| 58 | soil 14 | vs | soil 3 | 1 | 1.09200672 | 15.736597 | 0.7239678 | 0.031 |
| 59 | soil 14 | vs | soil 7 | 1 | 0.49712566 | 6.277985 | 0.5566584 | 0.026 |
| 60 | soil 14 | vs | soil 0 | 1 | 1.7097253 | 21.551373 | 0.782225 | 0.02 |
| 61 | soil 21 | vs | soil 3 | 1 | 1.30177148 | 19.64991 | 0.7660811 | 0.028 |
| 62 | soil 21 | vs | soil 7 | 1 | 0.6946835 | 9.21184 | 0.6481807 | 0.029 |
| 63 | soil 21 | vs | soil 0 | 1 | 1.71646096 | 22.529298 | 0.7896899 | 0.024 |
| 64 | soil 3 | vs | soil 7 | 1 | 0.15351121 | 4.575134 | 0.4778141 | 0.025 |
| 65 | soil 3 | vs | soil 0 | 1 | 1.83557513 | 44.438741 | 0.8810438 | 0.028 |
| 66 | soil 7 | vs | soil 0 | 1 | 1.56299104 | 34.36576 | 0.8729861 | 0.024 |

Supplementary figure 2D statistics

Bray-stress=0.2101336

# PERMANOVA (999 iterations)

|  | Df | SumOfSqs | R2 | F | Pr(>F) |
| --- | --- | --- | --- | --- | --- |
| group | 1 | 1.9795 | 0.16648 | 16.6651 | 0.001 |
| time | 5 | 5.16 | 0.43395 | 8.6881 | 0.001 |
| Residual | 40 | 4.7513 | 0.39958 |  |  |
| Total | 46 | 11.8909 | 1 |  |  |

# ANOVA on dispersion

|  | Df | Sum Sq | Mean Sq | F | Pr(>F) |
| --- | --- | --- | --- | --- | --- |
| Groups | 11 | 0.28742 | 0.026129 | 2.2081 | 0.03718 |
| Residuals | 35 | 0.41417 | 0.011833 |  |  |

# Pairwise Adonis

|  | pairs | Df | SumsOfSqs | F.Model | R2 | p.value | p.adjusted | sig |  |
| --- | --- | --- | --- | --- | --- | --- | --- | --- | --- |
| 1 | lake 1 | vs | lake 14 | 1 | 0.77269388 | 9.601507 | 0.6154218 | 0.029 | 1 |
| 2 | lake 1 | vs | lake 21 | 1 | 0.91248784 | 11.79355 | 0.6627992 | 0.036 | 1 |
| 3 | lake 1 | vs | lake 3 | 1 | 0.1592125 | 2.480326 | 0.29248 | 0.18 | 1 |
| 4 | lake 1 | vs | lake 7 | 1 | 0.53826247 | 7.18045 | 0.5447803 | 0.032 | 1 |
| 5 | lake 1 | vs | lake 0 | 1 | 1.50774645 | 23.539856 | 0.7968846 | 0.032 | 1 |
| 6 | lake 1 | vs | soil 1 | 1 | 0.95198146 | 6.481576 | 0.5192915 | 0.026 | 1 |
| 7 | lake 1 | vs | soil 14 | 1 | 0.99566221 | 12.691137 | 0.6789922 | 0.028 | 1 |
| 8 | lake 1 | vs | soil 21 | 1 | 0.98742666 | 12.823675 | 0.6812525 | 0.034 | 1 |
| 9 | lake 1 | vs | soil 3 | 1 | 1.20569841 | 16.764381 | 0.7364304 | 0.029 | 1 |
| 10 | lake 1 | vs | soil 7 | 1 | 0.9540407 | 11.839198 | 0.7030737 | 0.022 | 1 |
| 11 | lake 1 | vs | soil 0 | 1 | 1.59757245 | 24.883907 | 0.8057241 | 0.033 | 1 |
| 12 | lake 14 | vs | lake 21 | 1 | 0.12216345 | 3.675659 | 0.3798872 | 0.029 | 1 |
| 13 | lake 14 | vs | lake 3 | 1 | 0.91850575 | 45.801204 | 0.8841726 | 0.028 | 1 |
| 14 | lake 14 | vs | lake 7 | 1 | 0.19511649 | 6.329558 | 0.5133645 | 0.034 | 1 |
| 15 | lake 14 | vs | lake 0 | 1 | 1.41929945 | 71.26851 | 0.9223487 | 0.04 | 1 |
| 16 | lake 14 | vs | soil 1 | 1 | 0.86864132 | 8.454834 | 0.584914 | 0.028 | 1 |
| 17 | lake 14 | vs | soil 14 | 1 | 0.49961868 | 14.558769 | 0.7081537 | 0.023 | 1 |
| 18 | lake 14 | vs | soil 21 | 1 | 0.46492338 | 14.146755 | 0.7021853 | 0.037 | 1 |
| 19 | lake 14 | vs | soil 3 | 1 | 1.27840607 | 46.011867 | 0.8846417 | 0.031 | 1 |
| 20 | lake 14 | vs | soil 7 | 1 | 0.75040617 | 27.168895 | 0.8445704 | 0.026 | 1 |
| 21 | lake 14 | vs | soil 0 | 1 | 1.25718604 | 62.655501 | 0.9126071 | 0.029 | 1 |
| 22 | lake 21 | vs | lake 3 | 1 | 1.19428827 | 70.460973 | 0.9215286 | 0.035 | 1 |
| 23 | lake 21 | vs | lake 7 | 1 | 0.43050434 | 15.529505 | 0.7213127 | 0.039 | 1 |
| 24 | lake 21 | vs | lake 0 | 1 | 1.50937841 | 89.789026 | 0.9373623 | 0.027 | 1 |
| 25 | lake 21 | vs | soil 1 | 1 | 0.85831793 | 8.614668 | 0.5894536 | 0.025 | 1 |
| 26 | lake 21 | vs | soil 14 | 1 | 0.74242573 | 23.785916 | 0.7985625 | 0.031 | 1 |
| 27 | lake 21 | vs | soil 21 | 1 | 0.65633002 | 22.054269 | 0.7861288 | 0.033 | 1 |
| 28 | lake 21 | vs | soil 3 | 1 | 1.38611331 | 56.164045 | 0.9034812 | 0.029 | 1 |
| 29 | lake 21 | vs | soil 7 | 1 | 0.91616092 | 38.341759 | 0.8846378 | 0.034 | 1 |
| 30 | lake 21 | vs | soil 0 | 1 | 0.97333538 | 57.388322 | 0.9053453 | 0.03 | 1 |
| 31 | lake 3 | vs | lake 7 | 1 | 0.62895541 | 43.256639 | 0.878189 | 0.033 | 1 |
| 32 | lake 3 | vs | lake 0 | 1 | 1.31795732 | 363.207379 | 0.983749 | 0.032 | 1 |
| 33 | lake 3 | vs | soil 1 | 1 | 1.17087668 | 13.543528 | 0.692993 | 0.02 | 1 |
| 34 | lake 3 | vs | soil 14 | 1 | 1.18978459 | 65.984711 | 0.916649 | 0.03 | 1 |
| 35 | lake 3 | vs | soil 21 | 1 | 1.21564282 | 73.327976 | 0.9243646 | 0.031 | 1 |
| 36 | lake 3 | vs | soil 3 | 1 | 1.12361619 | 97.721786 | 0.9421529 | 0.033 | 1 |
| 37 | lake 3 | vs | soil 7 | 1 | 0.86254206 | 106.794377 | 0.955275 | 0.029 | 1 |
| 38 | lake 3 | vs | soil 0 | 1 | 1.80712933 | 478.215784 | 0.9876088 | 0.03 | 1 |
| 39 | lake 7 | vs | lake 0 | 1 | 1.17683816 | 81.720759 | 0.9316011 | 0.031 | 1 |
| 40 | lake 7 | vs | soil 1 | 1 | 0.92060788 | 9.468847 | 0.6121236 | 0.026 | 1 |
| 41 | lake 7 | vs | soil 14 | 1 | 0.36438493 | 12.650815 | 0.6782982 | 0.036 | 1 |
| 42 | lake 7 | vs | soil 21 | 1 | 0.3952681 | 14.452099 | 0.7066316 | 0.034 | 1 |
| 43 | lake 7 | vs | soil 3 | 1 | 1.12929374 | 50.708795 | 0.8941963 | 0.03 | 1 |
| 44 | lake 7 | vs | soil 7 | 1 | 0.5001244 | 23.811892 | 0.8264605 | 0.03 | 1 |
| 45 | lake 7 | vs | soil 0 | 1 | 1.57850344 | 108.481045 | 0.9475896 | 0.032 | 1 |
| 46 | lake 0 | vs | soil 1 | 1 | 1.30333264 | 15.099988 | 0.7156396 | 0.026 | 1 |
| 47 | lake 0 | vs | soil 14 | 1 | 1.16338761 | 65.023323 | 0.9155207 | 0.036 | 1 |
| 48 | lake 0 | vs | soil 21 | 1 | 1.16760314 | 71.027301 | 0.9221055 | 0.021 | 1 |
| 49 | lake 0 | vs | soil 3 | 1 | 1.36206275 | 119.913101 | 0.9523481 | 0.027 | 1 |
| 50 | lake 0 | vs | soil 7 | 1 | 0.92137115 | 116.490299 | 0.9588445 | 0.028 | 1 |
| 51 | lake 0 | vs | soil 0 | 1 | 1.53899151 | 422.853957 | 0.9860092 | 0.029 | 1 |
| 52 | soil 1 | vs | soil 14 | 1 | 0.7114963 | 7.064379 | 0.5407359 | 0.023 | 1 |
| 53 | soil 1 | vs | soil 21 | 1 | 0.75524493 | 7.608525 | 0.5590999 | 0.024 | 1 |
| 54 | soil 1 | vs | soil 3 | 1 | 0.47493775 | 5.042715 | 0.4566554 | 0.115 | 1 |
| 55 | soil 1 | vs | soil 7 | 1 | 0.43521776 | 4.056142 | 0.4478885 | 0.123 | 1 |
| 56 | soil 1 | vs | soil 0 | 1 | 0.72108843 | 8.33978 | 0.5815835 | 0.03 | 1 |
| 57 | soil 14 | vs | soil 21 | 1 | 0.03899249 | 1.264293 | 0.1740421 | 0.355 | 1 |
| 58 | soil 14 | vs | soil 3 | 1 | 1.03182221 | 40.053187 | 0.8697159 | 0.028 | 1 |
| 59 | soil 14 | vs | soil 7 | 1 | 0.35244553 | 13.990105 | 0.736705 | 0.026 | 1 |
| 60 | soil 14 | vs | soil 0 | 1 | 1.4987662 | 83.070556 | 0.9326377 | 0.028 | 1 |
| 61 | soil 21 | vs | soil 3 | 1 | 1.22680445 | 50.468659 | 0.8937464 | 0.034 | 1 |
| 62 | soil 21 | vs | soil 7 | 1 | 0.48747452 | 20.788874 | 0.8061179 | 0.022 | 1 |
| 63 | soil 21 | vs | soil 0 | 1 | 1.30650008 | 78.756872 | 0.9292093 | 0.038 | 1 |
| 64 | soil 3 | vs | soil 7 | 1 | 0.1354281 | 7.804411 | 0.6095096 | 0.022 | 1 |
| 65 | soil 3 | vs | soil 0 | 1 | 1.76724886 | 153.553848 | 0.9623951 | 0.037 | 1 |
| 66 | soil 7 | vs | soil 0 | 1 | 1.43550607 | 177.448462 | 0.972595 | 0.034 | 1 |

Figure 4A statistics

# Shapiro-Wilk normality test LakeCom alone

| W= 0.95564 | p-value= 0.5839 |
| --- | --- |

# Shapiro-Wilk normality test LakeCom+SynCom

| W= 0.84085 | p-value= 0.01002 |
| --- | --- |

# Shapiro-Wilk normality test SynCom alone

| W= 0.87893 | p-value= 0.03735 |
| --- | --- |

# Levene's Test for Homogeneity of Variance (center = median)

| Df | F value | Pr(>F) |
| --- | --- | --- |
| 11 | 0.8268 | 0.6148 |
| 36 |  |  |

# Outliers

| Condition | Time | Sample | Phase | Per g cell | is.outlier | is.extreme |
| --- | --- | --- | --- | --- | --- | --- |
| SoilCom+SynCom | 7 | SYN+SOIL2 stained | 2 | 6220000 | TRUE | FALSE |

# Repeated measures ANOVA on ranked values

| Effect | DFn | DFd | F | p |
| --- | --- | --- | --- | --- |
| Condition | 2 | 9 | 10.867 | 4.00E-03 |
| Time | 3 | 27 | 470.791 | 2.06E-23 |
| Condition:Time | 6 | 27 | 13.645 | 4.45E-07 |

# Pairwise *t*-test (on ranked values), Holm’s correction

| Time | .y. | group1 | group2 | n1 | n2 | p | p.adj | p.adj.signif |
| --- | --- | --- | --- | --- | --- | --- | --- | --- |
| 3 | ranked value | soilcom | syncom | 4 | 4 | 0.427 | 0.427 | ns |
| 3 | ranked value | soilcom | soilcom+syncom | 4 | 4 | 0.0479 | 0.0958 | ns |
| 3 | ranked value | syncom | soilcom+syncom | 4 | 4 | 0.0123 | 0.0369 | * |
| 7 | ranked value | soilcom | syncom | 4 | 4 | 0.382 | 0.382 | ns |
| 7 | ranked value | soilcom | soilcom+syncom | 4 | 4 | 0.0195 | 0.0586 | ns |
| 7 | ranked value | syncom | soilcom+syncom | 4 | 4 | 0.0876 | 0.175 | ns |
| 21 | ranked value | soilcom | syncom | 4 | 4 | 0.000515 | 1.03E-03 | ** |
| 21 | ranked value | soilcom | soilcom+syncom | 4 | 4 | 0.0043 | 4.30E-03 | ** |
| 21 | ranked value | syncom | soilcom+syncom | 4 | 4 | 0.00000812 | 2.44E-05 | **** |
| 60 | ranked value | soilcom | syncom | 4 | 4 | 0.00000493 | 1.48E-05 | **** |
| 60 | ranked value | soilcom | soilcom+syncom | 4 | 4 | 0.000199 | 3.98E-04 | *** |
| 60 | ranked value | syncom | soilcom+syncom | 4 | 4 | 0.00568 | 5.68E-03 | ** |

# Residuals

# Shapiro-Wilk normality test

| W=0.9592 | p-value=0.09373 |
| --- | --- |

# Levene's Test for Homogeneity of Variance (center = median)

| Df | F value | Pr(>F) |
| --- | --- | --- |
| 11 | 0.8268 | 0.6148 |
| 36 |  |  |

Figure 4B statistics

# Shapiro-Wilk normality test SoilCom alone

| W= 0.88725 | p-value= 0.0504 |
| --- | --- |

# Shapiro-Wilk normality test SoilCom+SynCom

| W= 0.92402 | p-value= 0.1957 |
| --- | --- |

# Shapiro-Wilk normality test SynCom alone

| W= 0.87893 | p-value= 0.03735 |
| --- | --- |

# Levene's Test for Homogeneity of Variance (center = median)

| Df | F value | Pr(>F) |
| --- | --- | --- |
| 11 | 0.9754 | 0.4854 |
| 36 |  |  |

# Outliers

| Condition | Time | Sample | Phase | Per g cell | is.outlier | is.extreme |
| --- | --- | --- | --- | --- | --- | --- |
| LakeCom | 21 | LA4 stained | 2 | 23500000 | TRUE | FALSE |
| SynCom | 21 | SA3 stained | 2 | 19600000 | TRUE | FALSE |
| SynCom+LakeCom | 21 | SL4 stained | 2 | 18900000 | TRUE | FALSE |

# Repeated measures two-way ANOVA on ranked values

| Effect | DFn | DFd | F | p | ges |
| --- | --- | --- | --- | --- | --- |
| Condition | 2 | 9 | 1.87E+01 | 6.20E-04 | 0.717 |
| Time | 3 | 27 | 5.11E+01 | 2.91E-11 | 0.689 |
| Condition:Time | 6 | 27 | 5.54E+01 | 6.52E-14 | 0.828 |

# Pairwise *t*-test (on ranked values), Holm’s correction

| Time | .y. | group1 | group2 | n1 | n2 | p | p.adj | p.adj.signif |
| --- | --- | --- | --- | --- | --- | --- | --- | --- |
| 3 | ranked value | lakecom | syncom | 4 | 4 | 1.32E-07 | 3.95E-07 | **** |
| 3 | ranked value | lakecom | lake+syn | 4 | 4 | 0.00000261 | 5.22E-06 | **** |
| 3 | ranked value | syncom | lake+syn | 4 | 4 | 0.00185 | 1.85E-03 | ** |
| 7 | ranked value | lakecom | syncom | 4 | 4 | 8.01E-08 | 2.40E-07 | **** |
| 7 | ranked value | lakecom | lake+syn | 4 | 4 | 0.00000021 | 4.20E-07 | **** |
| 7 | ranked value | syncom | lake+syn | 4 | 4 | 0.135 | 0.135 | ns |
| 21 | ranked value | lakecom | syncom | 4 | 4 | 0.527 | 1 | ns |
| 21 | ranked value | lakecom | lake+syn | 4 | 4 | 0.844 | 1 | ns |
| 21 | ranked value | syncom | lake+syn | 4 | 4 | 0.412 | 1 | ns |
| 60 | ranked value | lakecom | syncom | 4 | 4 | 0.00186 | 0.00372 | ** |
| 60 | ranked value | lakecom | lake+syn | 4 | 4 | 0.572 | 0.572 | ns |
| 60 | ranked value | syncom | lake+syn | 4 | 4 | 0.000811 | 0.00243 | ** |

# Residuals

# Shapiro-Wilk normality test

| W=0.97191 | p-value=0.3 |
| --- | --- |

# Levene's Test for Homogeneity of Variance (center = median)

| Df | F value | Pr(>F) |
| --- | --- | --- |
| 11 | 0.9754 | 0.4854 |
| 36 |  |  |

Figure 4C statistics

# *Day 0 removed from analysis.*

# Shapiro-Wilk normality test LakeCom

| W= 0.31233 | p-value= 8.371e-08 |
| --- | --- |

# Shapiro-Wilk normality test LakeCom_SynCom

| W= 0.82 | p-value= 0.005084 |  |
| --- | --- | --- |

# Shapiro-Wilk normality test SynCom

| W= 0.86421 | p-value= 0.0222 |  |  |  |  |  |
| --- | --- | --- | --- | --- | --- | --- |

# Shapiro-Wilk normality test SoilCom

|  |  |  |  |  | W= 0.67745 | p-value= 9.626e-05 |
| --- | --- | --- | --- | --- | --- | --- |

# Shapiro-Wilk normality test SoilCom_SynCom

| W= 0.79923 | p-value= 0.003594 |
| --- | --- |

# Levene's Test for Homogeneity of Variance (center = median), ranked value

| Df | F value | Pr(>F) |
| --- | --- | --- |
| 19 | 1.2213 | 0.272 |
| 60 |  |  |

# Outliers

| Condition | Time | Sample | Phase | Ranked value | is.outlier | is.extreme |
| --- | --- | --- | --- | --- | --- | --- |
| LakeCom | 3 | LA3 | 2 | 30 | TRUE | FALSE |
| LakeCom | 60 | LA2 | 2 | 22 | TRUE | FALSE |
| SoilCom | 60 | NA3 | 2 | 8 | TRUE | FALSE |
| LakeCom SynCom | 3 | SA2 | 2 | 64 | TRUE | FALSE |

# Repeated measures two-way ANOVA on ranked values

| Effect | DFn | DFd | F | p | ges |
| --- | --- | --- | --- | --- | --- |
| Condition | 4 | 15 | 346.736 | 1.40E-14 | 0.951 |
| Time | 3 | 45 | 71.274 | 3.99E-17 | 0.79 |
| Condition:Time | 12 | 45 | 8.477 | 4.45E-08 | 0.641 |

# Pairwise *t*-test (on ranked values), Holm’s correction

| Time | .y. | group1 | group2 | n1 | n2 | p | p.adj | p.adj.signif |  |
| --- | --- | --- | --- | --- | --- | --- | --- | --- | --- |
| 3 | ranked value | lakecom | soilcom | 4 | 4 | 3.30E-06 | 1.98E-05 | **** |  |
| 3 | ranked value | lakecom | syncom | 4 | 4 | 2.40E-10 | 2.40E-09 | **** |  |
| 3 | ranked value | soilcom | syncom | 4 | 4 | 1.56E-06 | 1.09E-05 | **** |  |
| 3 | ranked value | lakecom | syn+lake | 4 | 4 | 8.08E-08 | 6.46E-07 | **** |  |
| 3 | ranked value | soilcom | syn+lake | 4 | 4 | 2.54E-02 | 5.08E-02 | ns |  |
| 3 | ranked value | syncom | syn+lake | 4 | 4 | 1.22E-04 | 6.08E-04 | *** |  |
| 3 | ranked value | lakecom | syn+soil | 4 | 4 | 4.06E-08 | 3.65E-07 | **** |  |
| 3 | ranked value | soilcom | syn+soil | 4 | 4 | 8.94E-03 | 2.68E-02 | * |  |
| 3 | ranked value | syncom | syn+soil | 4 | 4 | 3.35E-04 | 1.34E-03 | ** |  |
| 3 | ranked value | syn+lake | syn+soil | 4 | 4 | 6.11E-01 | 6.11E-01 | ns |  |
| 7 | ranked value | lakecom | soilcom | 4 | 4 | 1.94E-05 | 1.17E-04 | *** |  |
| 7 | ranked value | lakecom | syncom | 4 | 4 | 5.00E-10 | 5.00E-09 | **** |  |
| 7 | ranked value | soilcom | syncom | 4 | 4 | 1.01E-06 | 7.05E-06 | **** |  |
| 7 | ranked value | lakecom | syn+lake | 4 | 4 | 1.58E-08 | 1.26E-07 | **** |  |
| 7 | ranked value | soilcom | syn+lake | 4 | 4 | 2.42E-04 | 9.69E-04 | *** |  |
| 7 | ranked value | syncom | syn+lake | 4 | 4 | 7.08E-03 | 2.12E-02 | * |  |
| 7 | ranked value | lakecom | syn+soil | 4 | 4 | 1.29E-08 | 1.16E-07 | **** |  |
| 7 | ranked value | soilcom | syn+soil | 4 | 4 | 1.77E-04 | 8.84E-04 | *** |  |
| 7 | ranked value | syncom | syn+soil | 4 | 4 | 9.83E-03 | 2.12E-02 | * |  |
| 7 | ranked value | syn+lake | syn+soil | 4 | 4 | 8.74E-01 | 8.74E-01 | ns |  |
| 21 | ranked value | lakecom | soilcom | 4 | 4 | 4.37E-08 | 1.75E-07 | **** |  |
| 21 | ranked value | lakecom | syncom | 4 | 4 | 2.15E-14 | 2.15E-13 | **** |  |
| 21 | ranked value | soilcom | syncom | 4 | 4 | 1.42E-11 | 1.27E-10 | **** |  |
| 21 | ranked value | lakecom | syn+lake | 4 | 4 | 9.12E-10 | 5.47E-09 | **** |  |
| 21 | ranked value | soilcom | syn+lake | 4 | 4 | 4.57E-03 | 9.15E-03 | ** |  |
| 21 | ranked value | syncom | syn+lake | 4 | 4 | 2.59E-10 | 1.81E-09 | **** |  |
| 21 | ranked value | lakecom | syn+soil | 4 | 4 | 5.39E-11 | 4.31E-10 | **** |  |
| 21 | ranked value | soilcom | syn+soil | 4 | 4 | 1.39E-05 | 4.16E-05 | **** |  |
| 21 | ranked value | syncom | syn+soil | 4 | 4 | 6.03E-09 | 3.02E-08 | **** |  |
| 21 | ranked value | syn+lake | syn+soil | 4 | 4 | 9.25E-03 | 9.25E-03 | ** |  |
| 60 | ranked value | lakecom | soilcom | 4 | 4 | 2.35E-01 | 3.11E-01 | ns |  |
| 60 | ranked value | lakecom | syncom | 4 | 4 | 1.91E-11 | 1.91E-10 | **** |  |
| 60 | ranked value | soilcom | syncom | 4 | 4 | 5.39E-11 | 4.85E-10 | **** |  |
| 60 | ranked value | lakecom | syn+lake | 4 | 4 | 1.13E-02 | 4.51E-02 | * |  |
| 60 | ranked value | soilcom | syn+lake | 4 | 4 | 1.20E-01 | 3.11E-01 | ns |  |
| 60 | ranked value | syncom | syn+lake | 4 | 4 | 2.42E-10 | 1.94E-09 | **** |  |
| 60 | ranked value | lakecom | syn+soil | 4 | 4 | 3.34E-04 | 2.00E-03 | ** |  |
| 60 | ranked value | soilcom | syn+soil | 4 | 4 | 4.10E-03 | 2.05E-02 | * |  |
| 60 | ranked value | syncom | syn+soil | 4 | 4 | 1.39E-09 | 9.70E-09 | **** |  |
| 60 | ranked value | syn+lake | syn+soil | 4 | 4 | 1.04E-01 | 3.11E-01 | ns |  |

# Residuals

# Shapiro-Wilk normality test

| W=0.91858 | p-value=2.834e-07 |
| --- | --- |

# Levene's Test for Homogeneity of Variance (center = median)

| Df | F value | Pr(>F) |
| --- | --- | --- |
| 19 | 1.2213 | 0.272 |
| 60 |  |  |

Figure 5A statistics

Bray-stress= 0.1492595

# PERMANOVA (999 iterations)

|  | Df | SumOfSqs | R2 | F | Pr(>F) |
| --- | --- | --- | --- | --- | --- |
| Condition | 3 | 7.3402 | 0.40249 | 32.895 | 0.001 |
| Time | 4 | 5.5417 | 0.30387 | 18.626 | 0.001 |
| Residual | 72 | 5.3553 | 0.29365 |  |  |
| Total | 79 | 18.2372 | 1 |  |  |

# ANOVA on dispersion

|  | Df | Sum Sq | Mean Sq | F | Pr(>F) |
| --- | --- | --- | --- | --- | --- |
| Groups | 19 | 0.043758 | 0.0023031 | 0.8515 | 0.6399 |
| Residuals | 60 | 0.162291 | 0.0027048 |  |  |

# Pairwise Adonis

| Pairs | Df | SumsOfSqs | F.Model | R2 | p.value | p.adjusted | sig |
| --- | --- | --- | --- | --- | --- | --- | --- |
| lake vs. soil | 1 | 3.923232 | 42.275475 | 0.52663002 | 0.001 | 0.006 | * |
| lake vs. lake+syn | 1 | 1.592763 | 10.880767 | 0.22259812 | 0.001 | 0.006 | * |
| lake vs. soil+syn | 1 | 3.850563 | 32.930841 | 0.46426689 | 0.001 | 0.006 | * |
| soil vs. lake+syn | 1 | 2.636311 | 15.522889 | 0.29002337 | 0.001 | 0.006 | * |
| soil vs. soil+syn | 1 | 0.557155 | 3.968929 | 0.09456826 | 0.012 | 0.012 | . |
| lake+syn vs. soil+syn | 1 | 2.120358 | 10.931881 | 0.22341020 | 0.001 | 0.006 | * |

Supplementary figure 5A statistics

| day | test type | Lake ctrl | dataset2 | statistic | p value | p value adjusted |
| --- | --- | --- | --- | --- | --- | --- |
| 3 | Levene's Test | Lake ctrl | Lake+Syn | 281.89974 | 6.49E-59 | 3.89E-58 |
| 3 | Levene's Test | Lake ctrl | Soil ctrl | 4.3526389 | 0.03711331 | 0.07422663 |
| 3 | Levene's Test | Lake ctrl | Soil+Syn | 247.879104 | 4.35E-52 | 2.17E-51 |
| 3 | Levene's Test | Lake+Syn | Soil ctrl | 186.236975 | 3.31E-40 | 1.33E-39 |
| 3 | Levene's Test | Lake+Syn | Soil+Syn | 1.12130096 | 0.28979154 | 0.28979154 |
| 3 | Levene's Test | Soil ctrl | Soil+Syn | 162.145736 | 2.98E-35 | 8.93E-35 |
| 3 | WilcoxonTest | Lake ctrl | Lake+Syn | 516782 | 2.52E-21 | 1.26E-20 |
| 3 | PairwiseWilcoxon | Lake ctrl | Soil ctrl | 340327 | 1.17E-05 | 2.35E-05 |
| 3 | PairwiseWilcoxon | Lake ctrl | Soil+Syn | 430430 | 3.16E-27 | 1.89E-26 |
| 3 | PairwiseWilcoxon | Lake+Syn | Soil ctrl | 263331 | 5.66E-09 | 1.70E-08 |
| 3 | PairwiseWilcoxon | Lake+Syn | Soil+Syn | 361107 | 0.11181456 | 0.11181456 |
| 3 | PairwiseWilcoxon | Soil ctrl | Soil+Syn | 309001 | 7.51E-13 | 3.00E-12 |
| 0 | Levene's Test | Lake ctrl | Lake+Syn | 6.51508426 | 0.01075161 | 0.03082406 |
| 0 | Levene's Test | Lake ctrl | Soil ctrl | 21.6695017 | 3.40E-06 | 1.70E-05 |
| 0 | Levene's Test | Lake ctrl | Soil+Syn | 0.07970038 | 0.77772594 | 0.77772594 |
| 0 | Levene's Test | Lake+Syn | Soil ctrl | 46.4324229 | 1.21E-11 | 7.23E-11 |
| 0 | Levene's Test | Lake+Syn | Soil+Syn | 6.59733545 | 0.01027469 | 0.03082406 |
| 0 | Levene's Test | Soil ctrl | Soil+Syn | 15.7315679 | 7.52E-05 | 0.00030095 |
| 0 | PairwiseWilcoxon | Lake ctrl | Lake+Syn | 896183 | 0.35951765 | 1 |
| 0 | PairwiseWilcoxon | Lake ctrl | Soil ctrl | 842160 | 0.9784643 | 1 |
| 0 | PairwiseWilcoxon | Lake ctrl | Soil+Syn | 871225 | 0.6532616 | 1 |
| 0 | PairwiseWilcoxon | Lake+Syn | Soil ctrl | 653211 | 0.34270643 | 1 |
| 0 | PairwiseWilcoxon | Lake+Syn | Soil+Syn | 677436 | 0.64455848 | 1 |
| 0 | PairwiseWilcoxon | Soil ctrl | Soil+Syn | 661779 | 0.74181984 | 1 |
| 60 | Levene's Test | Lake ctrl | Lake+Syn | 250.838893 | 2.00E-53 | 1.20E-52 |
| 60 | Levene's Test | Lake ctrl | Soil ctrl | 32.1031074 | 1.72E-08 | 3.44E-08 |
| 60 | Levene's Test | Lake ctrl | Soil+Syn | 145.362181 | 5.45E-32 | 2.72E-31 |
| 60 | Levene's Test | Lake+Syn | Soil ctrl | 83.2048632 | 1.95E-19 | 7.81E-19 |
| 60 | Levene's Test | Lake+Syn | Soil+Syn | 3.75850519 | 0.05271674 | 0.05271674 |
| 60 | Levene's Test | Soil ctrl | Soil+Syn | 38.8292366 | 6.45E-10 | 1.93E-09 |
| 60 | PairwiseWilcoxon | Lake ctrl | Lake+Syn | 707191 | 1.07E-34 | 4.29E-34 |
| 60 | PairwiseWilcoxon | Lake ctrl | Soil ctrl | 411464 | 2.37E-16 | 4.74E-16 |
| 60 | PairwiseWilcoxon | Lake ctrl | Soil+Syn | 154044 | 9.12E-30 | 2.74E-29 |
| 60 | PairwiseWilcoxon | Lake+Syn | Soil ctrl | 326376 | 1.79E-05 | 1.79E-05 |
| 60 | PairwiseWilcoxon | Lake+Syn | Soil+Syn | 133210 | 1.98E-58 | 1.19E-57 |
| 60 | PairwiseWilcoxon | Soil ctrl | Soil+Syn | 83286 | 4.29E-48 | 2.15E-47 |
| 7 | Levene's Test | Lake ctrl | Lake+Syn | 171.370345 | 3.01E-37 | 1.51E-36 |
| 7 | Levene's Test | Lake ctrl | Soil ctrl | 0.0289012 | 0.86502889 | 0.86502889 |
| 7 | Levene's Test | Lake ctrl | Soil+Syn | 106.81414 | 2.97E-24 | 8.91E-24 |
| 7 | Levene's Test | Lake+Syn | Soil ctrl | 184.996865 | 5.54E-40 | 3.32E-39 |
| 7 | Levene's Test | Lake+Syn | Soil+Syn | 9.02625477 | 0.00270346 | 0.00540691 |
| 7 | Levene's Test | Soil ctrl | Soil+Syn | 115.300395 | 5.15E-26 | 2.06E-25 |
| 7 | PairwiseWilcoxon | Lake ctrl | Lake+Syn | 376475 | 7.62E-13 | 3.81E-12 |
| 7 | PairwiseWilcoxon | Lake ctrl | Soil ctrl | 428533 | 2.49E-32 | 1.49E-31 |
| 7 | PairwiseWilcoxon | Lake ctrl | Soil+Syn | 357023 | 7.85E-12 | 3.14E-11 |
| 7 | PairwiseWilcoxon | Lake+Syn | Soil ctrl | 341127 | 0.29756547 | 0.59513095 |
| 7 | PairwiseWilcoxon | Lake+Syn | Soil+Syn | 301942 | 0.47701135 | 0.59513095 |
| 7 | PairwiseWilcoxon | Soil ctrl | Soil+Syn | 296619 | 0.03426334 | 0.10279002 |
| 21 | Levene's Test | Lake ctrl | Lake+Syn | 299.522231 | 6.22E-63 | 3.11E-62 |
| 21 | Levene's Test | Lake ctrl | Soil ctrl | 33.8908293 | 7.07E-09 | 1.41E-08 |
| 21 | Levene's Test | Lake ctrl | Soil+Syn | 317.098373 | 3.46E-65 | 2.07E-64 |
| 21 | Levene's Test | Lake+Syn | Soil ctrl | 72.1962211 | 4.25E-17 | 1.28E-16 |
| 21 | Levene's Test | Lake+Syn | Soil+Syn | 1.35983753 | 0.24371834 | 0.24371834 |
| 21 | Levene's Test | Soil ctrl | Soil+Syn | 81.246727 | 7.53E-19 | 3.01E-18 |
| 21 | PairwiseWilcoxon | Lake ctrl | Lake+Syn | 763190 | 1.96E-37 | 1.17E-36 |
| 21 | PairwiseWilcoxon | Lake ctrl | Soil ctrl | 344766 | 2.52E-18 | 1.26E-17 |
| 21 | PairwiseWilcoxon | Lake ctrl | Soil+Syn | 353472 | 0.72992798 | 0.72992798 |
| 21 | PairwiseWilcoxon | Lake+Syn | Soil ctrl | 264570 | 3.20E-05 | 6.41E-05 |
| 21 | PairwiseWilcoxon | Lake+Syn | Soil+Syn | 297286 | 1.44E-17 | 5.74E-17 |
| 21 | PairwiseWilcoxon | Soil ctrl | Soil+Syn | 154680 | 3.26E-06 | 9.79E-06 |

Figure 5D statistics

# Shapiro-Wilk normality test

| SoilCom W=0.94553 | p-value=0.3043 |
| --- | --- |
| LakeCom W=0.91477 | p-value=0.07864 |
| SoilCom replicates W=0.93117 | p-value=0.05275 |
| LakeCom replicates W=0.92983 | p-value=0.04859 |

# Levene's Test for Homogeneity of Variance (center = median)

| Df | F value | Pr(>F) |
| --- | --- | --- |
| 19 | 1.1711 | 0.3029 |
| 80 |  |  |

# Outliers

| Comparison | Time | Replicate | Value | is.outlier | is.extreme |
| --- | --- | --- | --- | --- | --- |
| Lake sum | 3 | 4 | 16.1 | TRUE | FALSE |
| Lake sum | 7 | 1 | 30 | TRUE | FALSE |
| Lake sum | 60 | 1 | 35 | TRUE | FALSE |
| Lake sum rep | 21 | 3 | 0.823 | TRUE | FALSE |
| Lake sum rep | 60 | 1 | 8.78 | TRUE | FALSE |
| Soil sum | 3 | 3 | 12.7 | TRUE | FALSE |
| Soil sum rep | 60 | 3 | 19.1 | TRUE | FALSE |

# Repeated measures ANOVA on ranked values

| Effect | DFn | DFd | F | p | ges |
| --- | --- | --- | --- | --- | --- |
| Condition | 3 | 16 | 34.862 | 3.01E-07 | 0.752 |
| Time | 4 | 64 | 34.135 | 3.06E-15 | 0.534 |
| Condition:Time | 12 | 64 | 10.439 | 5.11E-11 | 0.512 |

# Pairwise t-test (on ranked values), Holm’s correction

| Time | .y. | group1 | group2 | n1 | n2 | p | p.adj | p.adj.signif |
| --- | --- | --- | --- | --- | --- | --- | --- | --- |
| 0 | value | LAKE_SUM | LAKE_SUM_REP | 4 | 6 | 3.66E-01 | 1.00E+00 | ns |
| 0 | value | LAKE_SUM | SOIL_SUM | 4 | 4 | 6.39E-01 | 1.00E+00 | ns |
| 0 | value | LAKE_SUM_REP | SOIL_SUM | 6 | 4 | 6.90E-01 | 1.00E+00 | ns |
| 0 | value | LAKE_SUM | SOIL_SUM_REP | 4 | 6 | 6.49E-01 | 1.00E+00 | ns |
| 0 | value | LAKE_SUM_REP | SOIL_SUM_REP | 6 | 6 | 1.39E-01 | 8.33E-01 | ns |
| 0 | value | SOIL_SUM | SOIL_SUM_REP | 4 | 6 | 3.38E-01 | 1.00E+00 | ns |
| 3 | value | LAKE_SUM | LAKE_SUM_REP | 4 | 6 | 3.29E-09 | 1.98E-08 | **** |
| 3 | value | LAKE_SUM | SOIL_SUM | 4 | 4 | 1.02E-02 | 2.04E-02 | * |
| 3 | value | LAKE_SUM_REP | SOIL_SUM | 6 | 4 | 2.81E-07 | 1.12E-06 | **** |
| 3 | value | LAKE_SUM | SOIL_SUM_REP | 4 | 6 | 9.60E-09 | 4.80E-08 | **** |
| 3 | value | LAKE_SUM_REP | SOIL_SUM_REP | 6 | 6 | 3.63E-01 | 3.63E-01 | ns |
| 3 | value | SOIL_SUM | SOIL_SUM_REP | 4 | 6 | 1.09E-06 | 3.28E-06 | **** |
| 7 | value | LAKE_SUM | LAKE_SUM_REP | 4 | 6 | 5.67E-06 | 3.40E-05 | **** |
| 7 | value | LAKE_SUM | SOIL_SUM | 4 | 4 | 2.21E-01 | 4.41E-01 | ns |
| 7 | value | LAKE_SUM_REP | SOIL_SUM | 6 | 4 | 8.01E-05 | 3.20E-04 | *** |
| 7 | value | LAKE_SUM | SOIL_SUM_REP | 4 | 6 | 7.16E-06 | 3.58E-05 | **** |
| 7 | value | LAKE_SUM_REP | SOIL_SUM_REP | 6 | 6 | 8.87E-01 | 8.87E-01 | ns |
| 7 | value | SOIL_SUM | SOIL_SUM_REP | 4 | 6 | 1.04E-04 | 3.20E-04 | *** |
| 21 | value | LAKE_SUM | LAKE_SUM_REP | 4 | 6 | 2.14E-06 | 1.28E-05 | **** |
| 21 | value | LAKE_SUM | SOIL_SUM | 4 | 4 | 2.05E-02 | 4.10E-02 | * |
| 21 | value | LAKE_SUM_REP | SOIL_SUM | 6 | 4 | 4.69E-04 | 1.88E-03 | ** |
| 21 | value | LAKE_SUM | SOIL_SUM_REP | 4 | 6 | 3.95E-05 | 1.98E-04 | *** |
| 21 | value | LAKE_SUM_REP | SOIL_SUM_REP | 6 | 6 | 9.46E-02 | 9.46E-02 | ns |
| 21 | value | SOIL_SUM | SOIL_SUM_REP | 4 | 6 | 1.32E-02 | 3.95E-02 | * |
| 60 | value | LAKE_SUM | LAKE_SUM_REP | 4 | 6 | 7.19E-06 | 4.31E-05 | **** |
| 60 | value | LAKE_SUM | SOIL_SUM | 4 | 4 | 1.77E-03 | 7.07E-03 | ** |
| 60 | value | LAKE_SUM_REP | SOIL_SUM | 6 | 4 | 2.85E-02 | 8.39E-02 | ns |
| 60 | value | LAKE_SUM | SOIL_SUM_REP | 4 | 6 | 4.98E-04 | 2.49E-03 | ** |
| 60 | value | LAKE_SUM_REP | SOIL_SUM_REP | 6 | 6 | 2.80E-02 | 8.39E-02 | ns |
| 60 | value | SOIL_SUM | SOIL_SUM_REP | 4 | 6 | 8.09E-01 | 8.09E-01 | ns |

# Residuals

# Shapiro-Wilk normality test

| W=0.97536 | p-value=0.05754 |
| --- | --- |

# Levene's Test for Homogeneity of Variance (center = median)

| Df | F value | Pr(>F) |
| --- | --- | --- |
| 19 | 0.7397 | 0.7673 |
| 80 |  |  |

Figure 7A statistics

# Only last day compared

# Shapiro-Wilk normality test

| W=0.94829 | p-value=0.4631 |
| --- | --- |

# Levene's Test for Homogeneity of Variance (center = median)

| Df | F value | Pr(>F) |
| --- | --- | --- |
| 3 | 2.7876 | 0.08619 |
| 12 |  |  |

# Outliers

| Condition | Time | Sample | CFU g | is.outlier | is.extreme |
| --- | --- | --- | --- | --- | --- |
| Syn2w | 28 | Ni 16 | 58700000 | TRUE | FALSE |
| Syn3m | 28 | Ni 8 | 11000000 | TRUE | FALSE |

# ANOVA

|  | Df | SumSq | MeanSq | F value | Pr(>F) |
| --- | --- | --- | --- | --- | --- |
| Condition | 3 | 1.96E+15 | 6.54E+14 | 6.324 | 0.0081 |
| Residuals | 12 | 1.24E+15 | 1.03E+14 |  |  |

# Pairwise *t*-test, Holm’s correction

| .y. | group1 | group2 | n1 | n2 | p | p.signif | p.adj | p.adj.signif |
| --- | --- | --- | --- | --- | --- | --- | --- | --- |
| CFU g | SoilCom+Syn2w | SoilCom+Syn3m | 4 | 4 | 0.53 | ns | 1 | ns |
| CFU g | SoilCom+Syn2w | Syn2w | 4 | 4 | 0.223 | ns | 0.668 | ns |
| CFU g | SoilCom+Syn3m | Syn2w | 4 | 4 | 0.534 | ns | 1 | ns |
| CFU g | SoilCom+Syn2w | Syn3m | 4 | 4 | 0.0175 | * | 0.0699 | ns |
| CFU g | SoilCom+Syn3m | Syn3m | 4 | 4 | 0.00527 | ** | 0.0263 | * |
| CFU g | Syn2w | Syn3m | 4 | 4 | 0.00164 | ** | 0.00984 | ** |

# Residuals

# Shapiro-Wilk normality test

| W=0.98116 | p-value=0.9721 |
| --- | --- |

# Levene's Test for Homogeneity of Variance (center = median)

| Df | F value | Pr(>F) |
| --- | --- | --- |
| 3 | 2.7876 | 0.08619 |
| 12 |  |  |

Figure 7B statistics

# Shapiro-Wilk normality test

| W=0.80334 | p-value=0.003018 |
| --- | --- |

# Kruskal-Wallis rank sum test

| chi-squared=11.912 | df=3 | p-value=0.007692 |
| --- | --- | --- |

# Dunn test, Holm’s adjustment

|  | SoilCom+Syn2w | SoilCom+Syn3m | Syn2w |
| --- | --- | --- | --- |
| SoilCom+Syn3m | 0.3069 | - | - |
| Syn2w | 0.6559 | 0.1879 | - |
| Syn3m | 0.2988 | 0.0038 | 0.3626 |

Figure 7C statistics

Bray-stress= 0.2337854

# Permutation test for adonis under reduced model

# Number of permutation: 999

|  | Df | SumOfSqs | R2 | F | Pr(>F) |
| --- | --- | --- | --- | --- | --- |
| condition | 4 | 17.351 | 0.59408 | 34.759 | 0.001 |
| Residual | 95 | 11.856 | 0.40592 |  |  |
| Total | 95 | 29.207 | 1 |  |  |

# ANOVA on dispersion

|  | Df | Sum Sq | Mean Sq | F | Pr(>F) |
| --- | --- | --- | --- | --- | --- |
| Groups | 4 | 2.7729 | 0.69322 | 17.426 | 9.40E-11 |
| Residuals | 95 | 3.7793 | 0.03978 |  |  |

# Pairwise Adonis

| pairs | SumOfSqs | F.Model | R2 | p.value | p.adjusted |
| --- | --- | --- | --- | --- | --- |
| Syn3m vs. SoilCom+Syn3m | 0.821285 | 8.459117 | 0.18207658 | 0.001 | 0.01 |
| Syn3m vs. Syn2w | 5.981387 | 364.943267 | 0.90569392 | 0.001 | 0.01 |
| Syn3m vs. SoilCom+Syn2w | 5.09522 | 89.332686 | 0.70156916 | 0.001 | 0.01 |
| Syn3m vs. SoilCom | 5.986696 | 36.02102 | 0.48663231 | 0.001 | 0.01 |
| SoilCom+Syn3m vs. Syn2w | 5.169615 | 53.295959 | 0.58377128 | 0.001 | 0.01 |
| SoilCom+Syn3m vs. SoilCom+Syn2w | 4.245157 | 30.841391 | 0.44800651 | 0.001 | 0.01 |
| SoilCom+Syn3m vs. SoilCom | 4.060587 | 16.452385 | 0.3021426 | 0.001 | 0.01 |
| Syn2w vs. SoilCom+Syn2w | 0.15041 | 2.641276 | 0.06498998 | 0.096 | 0.096 |
| Syn2w vs. SoilCom | 6.40396 | 38.552621 | 0.50360942 | 0.001 | 0.01 |
| SoilCom+Syn2w vs. SoilCom | 5.463732 | 26.425971 | 0.41017575 | 0.001 | 0.01 |

**Supplementary references**

1. Čaušević S, Tackmann J, Sentchilo V *et al.* Reproducible propagation of species-rich soil bacterial communities suggests robust underlying deterministic principles of community formation. *mSystems*. 2022;**7**:e00160-22 <https://doi.org/10.1128/msystems.00160-22>

2. McMurdie PJ, Holmes S. phyloseq: An R package for reproducible interactive analysis and graphics of microbiome census data. *PLoS one*. 2013;**8**:e61217 https://doi.org/10.1371/journal.pone.0061217

3. Lahti L, Sudarshan S. Microbiome R package. 2012-2019 https://doi.org/[10.18129/B9.bioc.microbiome](https://doi.org/doi:10.18129/B9.bioc.microbiome)

4. Oksanen J, Kindt R, Legendre P *et al.* The vegan package: Community ecology package. 2007;**10**:719 https://CRAN.R-project.org/package=vegan

5. McMurdie PJ. Biomformat: An interface package for the BIOM file format. *R package version* 1.36.0. 2022 https://doi.org/1[0.18129/B9.bioc.biomformat](https://doi.org/doi:10.18129/B9.bioc.biomformat)

6. Wickham H, Averick M, Bryan J, *et al*. Welcome to the tidyverse. *J Open Source Softw*. 2019;**4**:1686 <https://doi.org/10.21105/joss.01686>

7. Wickham H, Francois R, Henry L, *et al*. dplyr: A grammar of data manipulation. *R package version 114*. 2023 [https://dplyr.tidyverse.org](https://dplyr.tidyverse.org/).

8. Wickham H. Reshaping data with the reshape package. *J Stat Softw*. 2007;**21**:1-20 https://doi.org/[10.18637/jss.v021.i12](https://doi.org/10.18637/jss.v021.i12)

9. Templ M, Hron K, Filzmoser P. robCompositions: An R-package for robust statistical analysis of compositional data. *Compositional data analysis. Theory and applications*, Chichester (UK): John Wiley & Sons 341-55

10. Pohlert T. Pmcmrplus: Calculate pairwise multiple comparisons of mean rank sums extended. *R package version* 1.4.1.

11. Martinez Arbizu P. Pairwiseadonis: Pairwise multilevel comparison using adonis. *R package version 041*. 2017

12. Kassambara A. rstatix: Pipe-friendly framework for basic statistical tests. *R package version 072*. 2023

13. Lenth R, Singmann H, Love J *et al.* Emmeans: Estimated marginal means, aka least-squares means *R package* *version* 1.3.4. 2019

https://cran.r-project.org/package=emmeans

14. Xu S, Yu G. MicrobiotaProcess: An R package for analysis, visualization and biomarker discovery of microbiome. *R package version* 1.0.5.

15. Wickham H. ggplot2. *Wiley Interdiscip Rev: Comput Stat*. 2011;**3**:180-85 https://doi.org/10.1002/wics.147

16. Kassambara A. ggpubr: 'ggplot2' based publication ready plots. *R package version 060*. 2023

17. Pagès H AP, Gentleman R, DebRoy S. Biostrings: Efficient manipulation of biological strings. *R package version* 2.64.0. 2022 https://doi.org/[10.18129/B9.bioc.Biostrings](https://doi.org/doi:10.18129/B9.bioc.Biostrings)

18. Signorell A. DescTools: Tools for descriptive statistics. *R package version 09950*. 2023

19. Fox J, Weisberg S. *An R companion to applied regression*, Third edn. Thousand Oaks CA: Sage, 2019.

20. Hothorn T, Bretz F, Westfall P. Simultaneous inference in general parametric models. *Biometr J*. 2008;**50**:346-63 https://doi.org/10.1002/bimj.200810425

21. Hervé M. RVAideMemoire: Testing and plotting procedures for biostatistics. *R package version* 09-69. 2018

22. Balkay L. Fca_readfcs, matlab central file exchange. https://www.mathworks.com/matlabcentral/fileexchange/9608-fca_readfcs

23. Shenhav L, Thompson M, Joseph TA *et al.* FEAST: Fast expectation-maximization for microbial source tracking. *Nat Methods*. 2019;**16**:627-32 <https://doi.org/10.1038/s41592-019-0431-x>

24. Becht E, McInnes L, Healy J *et al.* Dimensionality reduction for visualizing single-cell data using UMAP. *Nat Biotechnol*. 2019;**37**:38-44 <https://doi.org/10.1038/nbt.4314>

25. Bezanson J, Edelman A, Karpinski S *et al.* Julia: A fresh approach to numerical computing. *SIAM Rev*. 2017;**59**:65-98 https://doi.org/10.1137/141000671

26. JuliaStats. Distances.Jl, a Julia package for evaluating distances (metrics) between vectors. [Computer software].2018. <https://github.com/JuliaStats/Distances.jl>

27. McInnes L, Healy J, Melville J. UMAP: Uniform manifold approximation and projection for dimension reduction *ArXiv e-prints*. 2018;1802.03426 https://doi.org/10.48550/arXiv.1802.03426

28. Van Rossum G, Drake FL. *Python 3 reference manual.* , Scotts Valley, CA: CreateSpace., 2009.

29. Waskom ML. Seaborn: Statistical data visualization. *J Open Source Softw*. 2021;**6**:3021 https://doi.org/10.21105/joss.03021

30. JuliaStats. Clustering.Jl: A Julia package for clustering data. [Computer software]. 2020 https://github.com/JuliaStats/Clustering.jl

31. Bonham K, Kayisire A, Luo A *et al.* Microbiome.Jl and biobakeryutils.Jl - Julia packages for working with microbial community data. *J Open Source Softw*. 2021;**6**:3876 <https://doi.org/10.21105/joss.03876>

32. Wirbel J, Zych K, Essex M *et al.* Microbiome meta-analysis and cross-disease comparison enabled by the siamcat machine learning toolbox. *Genome Biol*. 2021;**22**:93 <https://doi.org/10.1186/s13059-021-02306-1>
